# Development of Australian mental health guidelines for community sport

**DOI:** 10.1136/bjsports-2024-108749

**Published:** 2025-02-19

**Authors:** Stewart Anthony Vella, Caitlin Liddelow, Simon M Rice, Richard Keegan, Kate Hall, Mark A Jones, David Revalds Lubans, Samantha McLeod, Anthony David Okely, Lisa S Olive, Rosemary Purcell, Lindsey J Reece, Simon Rosenbaum, Matthew J Schweickle, Kelsey Singh, Damien Stewart, Leisl Stimpson, Jordan T Sutcliffe, Megan Teychenne, Courtney C Walton, Christian Swann

**Affiliations:** 1 Global Alliance for Mental Health and Sport, School of Psychology, University of Wollongong, Wollongong, New South Wales, Australia; 2 Institute of Men's Health, Movember, Richmond, Victoria, Australia; 3 Institute of Men's Health, Movember, Melbourne, Victoria, Australia; 4 Centre for Youth Mental Health, The University of Melbourne Faculty of Medicine Dentistry and Health Sciences, Melbourne, Victoria, Australia; 5 Research Institute for Sport and Exercise, University of Canberra, Canberra, Australian Capital Territory, Australia; 6 Australian Football League, Melbourne, Victoria, Australia; 7 SEED Lifespan Strategic Research Centre, School of Psychology, Deakin University Faculty of Health, Burwood, Victoria, Australia; 8 Football Australia, Sydney, New South Wales, Australia; 9 Centre for Active Living and Learning, College of Human and Social Futures, The University of Newcastle, Callaghan, New South Wales, Australia; 10 Hunter Medical Research Institute, New Lambton Heights, New South Wales, Australia; 11 The SAM Centre, Preston, Victoria, Australia; 12 APS College of Sport and Exercise Psychologists, Melbourne, Victoria, Australia; 13 Early Start, School of Health and Society, University of Wollongong, Wollongong, New South Wales, Australia; 14 Elite Sports and Mental Health, Orygen The National Centre of Excellence in Youth Mental Health, Parkville, Victoria, Australia; 15 Australian Sports Commission, Belconnen, Australian Capital Territory, Australia; 16 Discipline of Psychiatry and Mental Health, University of New South Wales, Sydney, New South Wales, Australia; 17 Disability Sports Australia, Sydney, New South Wales, Australia; 18 Room23 Psychology, Beerwah, Queensland, Australia; 19 Special Olympics Australia, Concord West, New South Wales, Australia; 20 Military Psychology and Leadership, Royal Military College of Canada, Kingston, Ontario, Canada; 21 Institute for Physical Activity and Nutrition (IPAN), School of Exercise and Nutrition Sciences, Deakin University, Geelong, Victoria, Australia; 22 Melbourne School of Psychological Sciences, University of Melbourne, Melbourne, Victoria, Australia; 23 Physical Activity, Sport and Exercise Research Theme, Faculty of Health, Southern Cross University, Coffs Harbour, New South Wales, Australia; 24 Manna Institute, Southern Cross University, Coffs Harbour, New South Wales, Australia

**Keywords:** Sports, Research, Psychology, Public health

## Abstract

**Objective:**

The need for clear and actionable guidelines for the promotion and protection of mental health in organised community sport has previously been identified. This study aimed to provide guidelines to promote and protect mental health in organised community sport in Australia.

**Methods:**

Guideline development was informed by (1) systematic reviews of the evidence pertaining to existing mental health guidelines in sport and mental health interventions in community sport; (2) an expert Delphi consensus study and (3) key stakeholder input via focus groups. A Guideline Development Committee comprising experts and key stakeholder representatives articulated nine distinct guidelines.

**Results:**

These guidelines address the areas of: mental health literacy training; mental health support pathways and processes; responding to mental health emergencies; responding to major events that may impact mental health; having a mental health plan in place; reducing stigmatising attitudes; appointing a dedicated mental health champion; coach education and promoting well-being within the organisation.

**Conclusions:**

We provide guidance for promoting and protecting mental health in community sport. Monitoring uptake and measuring the effectiveness of the guidelines are important areas of future work to advance positive mental health for everybody involved in community sport.

**WHAT IS ALREADY KNOWN ON THIS TOPIC**

- Organised community sports clubs have an important role to play in ensuring the mental health and well-being for the diverse range of individuals involved.
- While some state and national sports organisations have published guidelines to promote mental health and well-being, often these guidelines are not evidence-based.

**WHAT THIS STUDY ADDS**

- We present a world-first comprehensive, evidence-based set of nine actionable guidelines to protect and promote mental health and well-being in organised community sport in Australia.
- The guidelines focus on promoting well-being and reducing mental health stigma, increasing mental health literacy and locating appropriate help and resources, promoting psychologically safe coach development, having a dedicated mental health champion, developing a mental health action plan and responding to an acute mental health emergency and major events that may impact mental health.

**HOW THIS STUDY MIGHT AFFECT RESEARCH, PRACTICE OR POLICY**

- These guidelines complement and extend the current suite of policies at the highest level of sport governance in Australia.
- These guidelines can enhance the effectiveness of mental health initiatives by national sports organisations and government bodies in Australia by providing an overarching national approach, and thereby also extending guidance to those sports and geographies without any current guidance in place.

Over 13.5 million (53%) Australians participate in organised sport for recreation.^1^ In addition to the long-term benefits of sport participation on physical activity,^2^ there is a growing consensus that organised community sports clubs—defined as *those organised sports environments where sport is, or should be, the primary purpose of participation*—have an important and influential role to play in ensuring the mental health and well-being of the diverse range of individuals involved in community sport (eg, players, coaches, parents and volunteers). There is an estimated one in eight people (1 billion) globally reportedly currently living with a mental health condition.^3^ In Australia, suicide is the leading cause of death of young Australians (aged 16–24 years),^4^ and is also the leading contributor to the total burden of disease in Australia.^5^ However, many mental health problems are preventable.^6^ Therefore, it is imperative that mental health promotion initiatives are established in a range of community-based settings (eg, schools and workplaces) to establish a mentally healthy world.^7^ While most instances of mental health problems are likely to occur outside of the organised community sport environment, and participation is commonly associated with experiencing fewer mental health difficulties,^8 9^ this is not always the case.

On this basis, there have recently been calls for clear and actionable guidelines for the promotion and protection of mental health in organised community sport.^10^ In part, those calls have been actioned. For example, the Australian Football League (AFL) have a mental fitness charter in place,^11^ which includes a ‘quick win action plan’ for community AFL clubs. The Western Australian government have a Mental Health and Wellbeing Community Sport Framework,^12^ while the South Australian Government have a separate Mental Fitness Charter for Sport and Recreation^13^ and the Victorian Government have provided support for sports organisations in seeking mental health providers.^14^ Such responses have strengthened the capabilities of the sport system to act on mental health challenges experienced by those in sport, although many state and national sports organisations still lack explicit guidance (We define state sport organisations as the peak governing body sport in the different states and territories of Australia (eg, the Football West) National sport organisations are defined as the national governing bodies for specific sports in Australia, often having ties with the Australian Olympic Committee and/or the Australian Sports Commission (eg, Football Australia). National sport organisations aim to ensure the growth and integrity of the sport at a national level and provide elite athlete pathways.). As such, there is a need for a comprehensive national approach and guidance to promote mental health and well-being and prevent mental health problems in organised community sport participants and members.

Therefore, to meaningfully address mental health in organised community sport, a whole-of-system approach is needed.^15^ A system-based approach recognises the role of various national sports organisations and government bodies, each of whom may have their own mental health charters, as well as the role of community clubs and organisations, regional governing bodies and individuals such as coaches. Currently, the community sports sector has no such unifying or universal evidence-based strategy to address mental health and well-being in sport,^10^ despite generally strong community sport stakeholder support for mental health initiatives.^16 17^ Research has additionally shown that sports organisations report being under-resourced, undertrained and lack the knowledge to put in place even basic strategies to provide a safe and healthy environment for all participants.^16 18^ Many Australian community sports organisations have also expressed concerns regarding their ability to support long-term positive changes to mental health and well-being.^19^ Therefore, a necessary component of effective guidelines is that they are appropriate for the culture of sport, resources and programming available to sports clubs and organisations, and sports clubs’ preferences and capabilities regarding implementation.^20^

This paper outlines the processes undertaken to develop a set of Australian mental health guidelines for community sport, as well as the outcomes (ie, guidelines) of those processes. The aim of the project was to deliver mental health guidelines for the Australian community sport sector that are based on sector needs and preferences; acceptable and usable; able to be implemented by organised community sporting clubs and are effective in promoting mental health and well-being and preventing mental health problems.

## Methods

The protocol for the development of these guidelines followed the National Health and Medical Research Council ‘Guidelines for Guidelines’ recommendations and has been published elsewhere.^21^ Given the need to establish guidelines in the absence of any previous iterations, the broader research team first conducted two systematic reviews to compile, synthesise and evaluate all current mental health guidelines available in sport,^22^ and mental health interventions in community sport.^23^ An expert Guideline Development Committee was then convened, representing a range of key stakeholders in mental health and sport in Australia. Members of the expert Guideline Development Committee represented Sport Medicine Australia, Australian Sports Commission, Football Australia, the AFL, Australian Psychological Society, Disability Sport Australia and Exercise and Sports Science Australia. In addition, nine well-established clinicians and researchers with recognised expertise in mental health research within the clinical sport and exercise psychology sector, from around Australia, were also invited to join the committee. These experts were invited based on their published research on mental health and sport up to late 2020, with several of the experts identified via professional networks to which the principal investigators were connected to. The total number of expert Guideline Development Committee members was 16 (additional details about the Guideline Development Committee are reported elsewhere).^24^

### Patient and public involvement

In line with the Community-Based Participatory Research framework,^25^ each phase of this research engaged key stakeholders and/or experts in mental health and sport in Australia. Community sport members (ie, players, coaches, parents and officials) were involved in phases I and IV, and experts as part of the Guideline Development Committee were involved in the research via phases II, III and IV. The final Australian mental health guidelines for community sport are the result of several iterative phases with involvement from key stakeholder groups.

### Equity, diversity and inclusion statement

The key research team consists of one woman (lead researcher, early-career) and four men (senior researchers and mentors), and all members of the research team are from Australia due to the primary research aims focussing on the Australian sport environment. Experts on the Guideline Development Committee are mostly gender balanced (seven women and nine men) and represented key marginalised groups, such as people with disabilities. Expert representatives of other marginalised groups in Australia were invited to join the Guideline Development Committee but did not end up participating. Similarly, the focus groups in phase I were held virtually to allow for inclusivity of community sport organisations located regionally or in other Australian states. Participants in this phase were purposively sampled to ensure breadth in responses and generalisability of the final output. The participants in the focus groups included both women and men, of various ages and backgrounds, in a range of roles in their community sport organisation. The Australian mental health guidelines for community sport have been purposely designed to be flexible and adaptable to varying types of community sport organisations, regardless of sport type, geographical location, gender and culture breakdown and socioeconomic background. In the limitations section, we acknowledge issues with generalisability due to possible self-selection biases (ie, invested interest in mental health and well-being in sport) of participants and sport clubs in phase I.

### Preliminary phase: systematic reviews

The first systematic review synthesised and evaluated all existing sport-based mental health guidelines in the scientific literature. The methods, results and conclusions of this review have been published.^22^ In total, 13 distinct guidelines were included in the review that spanned Paralympic and Olympic athletes (n=1), elite athletes (n=5), competitive athletes (n=2), collegiate athletes (n=3), secondary school athletes (n=1) and practitioners (n=1). A meta-synthesis of the guideline recommendations was the primary outcome relevant to the development of these guidelines. The synthesis that was undertaken generated six major areas for the articulation of mental health guidelines in sport, namely (1) writing a mental health plan; (2) provision of mental healthcare; (3) athlete support system; (4) high-risk events; (5) athlete mental health and (6) future directions. These themes were used in phase III of the present study for the consideration of the expert Guideline Development Committee regarding their appropriateness and feasibility in the Australian community sport context.

The second systematic review synthesised and evaluated all existing mental health interventions in community sport that were available in the scientific literature. The methods, results and implications have been reported in detail elsewhere.^23^ Nineteen studies that were primarily psychoeducational in nature demonstrated small-to-medium beneficial effects on levels of anxiety, stress and well-being. Larger effects were demonstrated for stigmatising attitudes, knowledge about mental health and intention to provide help for mental health problems. The range and outcomes of effective interventions were used by the expert Guideline Development Committee in phase III when considering the articulation of specific guidelines.

### Phase I: focus groups with community sport stakeholders

To develop a robust understanding of the preferences for the content, purpose and scope of mental health guidelines for community sport, focus groups were conducted with community sport participants. In the last quarter of 2021, 31 community sport participants from around Australia, both metropolitan and regional locations, participated across nine focus groups. Focus groups were chosen as the preferred data collection method, compared with individual interviews, to develop a deeper understanding of the topic as well as generate a richer discussion between stakeholders who may not have thought of specific ideas individually.^26^ Participants represented players, committee members (club and association level), coaches and parents from a range of sports. Several participants held dual roles within their respective sport and/or club. A semistructured interview guide was developed, comprising three main questions: (1) Do you think mental health guidelines are needed in sport?; (2) What do you think should be covered in mental health guidelines for sport clubs? and (3) What specific content would you like to see as part of the proposed guidelines?

To support the articulation of draft guidelines in phase III, a series of key focus areas were identified following the nine stages of reflexive thematic analysis.^27^ To increase the trustworthiness of the data, members of the key research team also acted as ‘critical friends’ via peer debriefing throughout the period in which the focus groups were conducted. This method of trustworthiness encouraged the reflection on and exploration of alternative interpretations of the focus group data. After completing the analysis, the key focus areas identified in the data were presented to the Guideline Development Committee. The first focus area reflected the need for increased education in recognising and supporting mental health issues, and those experiencing them, as well as what the appropriate steps are for referring to resources and additional care. Second, participants emphasised the influence that mental health stigma still has in sport, and how it is not yet viewed as equally important as physical health. Third, the need for upskilling and additional training for coaches was discussed to ensure that coaches are as follows: (1) able to support the mental health of their players; and (2) able to coach in a psychologically safe manner to ensure that they are not negatively impacting their players. Fourth, many participants expressed that each level of sport management (eg, state organisations) has a role to play in ensuring the mental health and psychological safety of sport participants. Participants expressed a desire for having a ‘mental health champion’ in their club to facilitate and manage everything related to the mental health and well-being of members. Participants expressed how a top-down approach to implementation was necessary to ensure that local sport associations and clubs implemented the guidelines. They were required to be the ‘role models’ and engage in some sort of monitoring of clubs. Fifth, participants discussed potential barriers to the implementation of the guidelines, such as the need for the guidelines to be actionable and specific, offer different recommendations for different groups of members (eg, gender, age and culture) and to consider the lack of funding that sport clubs receive, and the lack of time and expertise that club volunteers possess.

### Phase II: Delphi study with experts

To complement the views of community sport participants, 21 experts (16 of which made up the Guideline Development Committee) were convened to provide input into the potential scope and content of the mental health guidelines. The aim of this phase was to explore and synthesise the opinions of those experts using the Delphi technique. This phase has been published in detail elsewhere.^24^ An initial round of open-ended questions was used to generate the major areas relevant to mental health protection and promotion in Australian community sport, as well as the perceived responsibilities of various levels of organisations (eg, clubs, regional associations and governing bodies). The qualitative responses from this round of questions were synthesised and 13 key statements were generated. The statements were related to the provision of mental health promotion, prevention and care in community sport. More specifically, eight of the statements related to the role of community sport organisations in the promotion of positive mental health, and the prevention of mental health problems. An additional five statements related to the responsibilities of the different levels of sport governance (ie, regional, state and national sporting organisations). Notably, these experts did not indicate that mental healthcare (ie, the care for people with mental health problems) was the responsibility of the community sport system, and as such, no statements were generated based on mental healthcare.

A second round of quantitative items was used to help reach consensus on each of the 13 statements that were generated. The statements were presented to the experts who were asked to rate the extent to which they agreed with each statement, and to provide a rating of its importance for inclusion in national mental health guidelines for community sport. Based on the responses of the expert committee, all 13 statements received consensus (ie, >80% of the committee in agreement, in line with published recommendations for consensus methods for guideline development)^28^ and were retained as important inclusions for mental health guidelines. These 13 statements were presented to the Guideline Development Committee during phase IV, in combination with the findings from the previous phases, as a basis for articulating mental health guidelines.

### Phase III: national consensus meeting and draft guideline writing

In February 2022, the Guideline Development Committee (16 experts from phase II) met in Wollongong, Australia for 2 days to consider the evidence and data accumulated during phases I–III and with the purpose to reach consensus on a set of mental health guidelines for community sport in Australia. The two systematic reviews, community sport participant data and expert Delphi data were used as the foundation for the articulation of the first draft of the guidelines. The evidence from these four foundational projects was synthesised by the research team and a series of topics/statements was presented to the expert Guideline Development Committee, whose role was to collaboratively: (1) consider the evidence for guideline development and ensure that the perspectives of end-user groups from phase I were integrated; (2) discuss, refine and finalise the scope and purpose of the guidelines and (3) develop a set of draft guidelines. Following discussion, each draft guideline was put to a confidential vote in real-time that was facilitated by Poll Everywhere. Initially, committee members voted for whether a specific area of interest (eg, mental health literacy (knowledge and beliefs about mental disorders which aid in their recognition, management or prevention^29^ training) should form part of the guidelines (see table 1 for an overview). If consensus was reached, the discussion then turned to the phrasing of a relevant guideline. After drafting the relevant guideline, a second vote was undertaken to assess the extent to which consensus had been reached on the articulation of the guideline. For both rounds of voting, a minimum of 80% agreement was required for consensus to be achieved. Where consensus was not reached in the first round of voting, or dissenting views were expressed, additional discussion was undertaken and a second vote cast. With the aid of collaborative discussion (ie, open discussions among the group), consensus was reached on the scope of all draft guidelines. For any remaining dissenting views (ie, for the guidelines that did not reach 100% consensus) further discussion was had to ensure that all members of the development committee were satisfied with the final guideline. Two members of the research team also took notes during the 2 day event to ensure trustworthiness of the data, and opportunities to check back on discussions, if required.

**Table 1:** Scope of the mental health guidelines and expert consensus levels

Topic area	Question	Yes agreement (%)
Mental health literacy training	Should mental health literacy training form part of the guidelines?	100
Referral pathways and procedures	Should there be a formal referral pathway from clubs to mental health services/support?	Round 1: 75Round 2: 88
Confidentiality	Should confidentiality of discussions and disclosure (unless presenting a risk to self or others) form part of the guidelines?	20
Mandatory reporting	Should mandatory reporting of risk/danger form part of the guidelines?	0
Stigmatising attitudes	Should there be a guideline around reducing mental health stigma in sport?	93
Coach education	Should there be a guideline around providing training/education/guidance on psychologically safe coaching?	100
Dedicated mental health roles	Should there be dedicated mental health roles in clubs/associations?	100
Mental health crises	Should there be a formal process for what to do in a mental health crisis?	100
Mental health plans	Should there be a concrete plan for how to address/promote mental health in clubs?	100
Well-being	Should there be a guideline related to actively promoting mental health and well-being in clubs?	100

Agreement refers to the percentage of the Guideline Development Committee members who responded ‘yes’ to the proposed question.

### Phase IV: feedback from stakeholders and experts

Following the consensus meeting, the guidelines document was drafted over the course of 6 months. Based on the discussions in phase III, the final document outlined each of the agreed-upon guidelines with additional information provided to assist in implementation: why is this guideline important, to whom does this guideline pertain to (eg, coaches and players), how should it be implemented, when should it be implemented and by whom should this guideline be managed? In October and November 2022, five follow-up focus groups were conducted with community sport participants (n=19) who participated in the original focus groups in phase I. Similar to phase I, the focus groups were facilitated virtually by two of the research team members. The participants of each focus group were emailed a copy of the draft guideline document in advance, and during the focus group were asked to provide feedback on each of the guidelines, as well as the accompanying guideline information. The focus groups were transcribed verbatim, and any recommended changes or actionable feedback related to the guidelines were noted.

On collating the feedback, where possible, recommended changes were made to the guidelines. The types of changes made were related to the use of language, the order in which the guidelines should appear, the inclusion of templates to assist in implementing some of the guidelines, providing recommended timelines, design and marketing of the future product and adding more information to specific guidelines. Two of the guidelines received specific feedback related to their similar wording, however, before the changes could be made, it was imperative that the Guideline Development Committee agreed with this change. In the last quarter of 2023, the Guideline Development Committee was consulted regarding the wording changes, and all members of the committee provided their approval. On making this change, the Guideline Development Committee received the final draft guidelines document for their review. No concerns were raised by the committee and thus the guidelines were considered ready for implementation.

## Results

### Scope of the guidelines

Nine topics were put forward to the Guideline Development Committee based on the evidence synthesised in the systematic reviews and phases I and II. These are presented in table 1, along with the specific question that was put to the committee and the level of consensus. Seven of the nine topic areas received consensus to move forward as a specific guideline, and two topic areas—‘confidentiality’, and ‘mandatory reporting’—did not reach consensus and were not included in guideline drafting. Members of the Guideline Development Committee advised that these two topics were covered in other topic areas (eg, referral pathways and mental health crises), and already formed part of existing sport policy and protocol in Australia. Similarly, the mental health crises guideline was deemed more important to include, as it incorporated notions of both confidentiality and mandatory reporting, in addition to detailed processes for dealing with an acute mental health crisis. The topic area of ‘referral pathways’ was the only topic that required two rounds of voting. Initially, the development committee expressed concern regarding the use of the word ‘formal’ and highlighted the importance of informal mental health support. The committee was also concerned over whether it was the responsibility of sport organisations to ‘refer’ members to mental health support services. After discussion and amendment of the wording of the possible guideline, the development committee voted again and the topic area received consensus to move forward. One topic area, ‘well-being’ was added to the list of potential guidelines after 2 days of in-depth discussions. The committee felt that while the promotion of mental well-being was somewhat covered by the current guidelines, it warranted its own guideline to ensure that sporting organisations/associations were not doing any harm. This inclusion received a 100% consensus vote.

Despite the ‘mental health crises’ topic area and guideline receiving consensus, there were additional discussions around the need to also have a guideline specifically for dealing with major events that may influence the mental health of sport organisations’ members. The Guideline Development Committee believed that it was important to delineate between an acute mental health crisis (eg, a situation in which a person’s mental health puts them at risk of harming themselves or others), versus an incident such as a critical incident or natural disaster that could negatively affect the mental health of members (eg, unexpected death of current/past member). As such, the development committee agreed to also include a guideline for handling such incidents and events (see table 2 for the draft guidelines), but this was not voted on as the scope/topic area level due to it being related to the crises topic area.

**Table 2:** Draft guidelines based on the topic areas that received consensus

Topic area	Approved guideline	Agreement (%)
Mental health literacy	At a minimum, community sport organisations need to demonstrate/evidence a commitment to enhancing the mental health literacy/education/awareness of their key stakeholders.	93
Referral pathways and processes	At a minimum, community sport organisations are aware/knowledgeable on what appropriate mental health services are available, and how to access them. If clubs want to do more, they can establish a relationship with a service provider.	100
Mental health crises	At a minimum, community sporting organisations need to have a plan for how to respond to an identified mental health crises.	100
Critical incidents	At a minimum, community sport organisations need to have a plan for how to respond to major events or critical incidents that may impact the psychological well-being of their members.	100
Mental health plans	At a minimum, community sport organisations need to have a completed, up-to-date mental health action plan outlining how they will prevent, promote and respond to mental health in sport	100
Stigmatising attitudes	At a minimum, community sport organisations demonstrate an ongoing commitment to promoting positive attitudes about mental health and well-being.	100
Coach education	At a minimum, community sport organisations need to support their coaches to participate in ongoing coach development in methods that promote safe/supportive coaching.	100
Dedicated mental health roles	At a minimum, community sport organisations need to have a mental health officer who actively oversees and contributes to the implementation of the mental health guidelines.	100
Well-being	At a minimum, community sport organisations demonstrate an ongoing commitment to promoting and optimising the well-being of the club community.	100

Agreement refers to the percentage of the Guideline Development Committee members who agreed with the draft guideline.

### The final guidelines

The final guidelines were accompanied by an explanation that the guidelines represent the minimum acceptable standards for a sport organisation to protect the mental health of its members and promote members’ well-being. This accompanying explanation emphasised that the guidelines were recommendations for best practice, not policy, and that they were flexible in their implementation based on the unique needs of the individual sports organisation, with respect to age, culture and gender, but also resources with regard to time, money and volunteers. For example, several of the guidelines were accompanied by different implementation options depending on what is feasible for the respective club, such as free or paid mental health literacy programmes, or having a subcommittee of mental health champions rather than a single individual. It was also emphasised that the guidelines were developed to complement and be implemented alongside other current governing guidelines, legislation and campaigns in Australian sport, such as the Member Protection Policy^30^ and Good Sports.^31^

The feedback from the expert panel and the community sport stakeholders in phase IV was collated, considered and implemented (if feasible). Some of the key areas of feedback concerned the wording of some of the guidelines being too ‘academic’, some of the accompanying content being too long, the need for templates to accompany some guidelines and suggestions for how to design the final document. The final names and wording of each guideline are presented in table 3. Please see online supplemental material for the full version of the guidelines, any accompanying information, and templates.

**Table 3:** Australian mental health guidelines for community sport

Title	Guideline
Promoting Well-being	Community sport organisations need to demonstrate a commitment to enhancing the well-being of everybody involved in the club.
Reducing Mental Health Stigma	Community sport organisations should demonstrate an ongoing commitment to promoting positive attitudes about mental health.
Promoting Coach Development	Community sport organisations need to support their coaches to participate in ongoing coach development in methods that promote safe/supportive coaching.
Increasing Mental Health Literacy	Community sport organisations need to demonstrate a commitment to enhancing the mental health literacy of their key stakeholders.
Locating Appropriate Help	Community sport organisations need to demonstrate an awareness on what appropriate mental health services are available, and how to access them.
Responding to a Mental Health Emergency	Community sport organisations should have a plan for how to respond to an identified mental health emergency (eg, risk or suicide or harm to self/others).
Responding to Major Events that May Impact Mental Health	Community sport organisations should have a plan for how to respond to major events or critical incidents that may impact the psychological well-being of their members.
Implementing a Mental Health Champion	Community sport organisations and clubs need to have a mental health champion who actively oversees and contributes to the implementation of the mental health guidelines.
Developing a Mental Health Action Plan	Community sport organisations should have a completed up-to-date mental health action plan outlining how they will prevent, promote and respond to mental health and well-being in sport.

## Discussion

This project addressed the need to provide evidence-based approaches to protect the mental health of over 13 million Australians who participate in organised sport, many of whom are children and adolescents.^1^ The resultant set of mental health guidelines for the Australian community sporting sector are a universal and unified approach and will assist community sport organisations to be evidence-informed in their approaches to mental health promotion and protection. The guidelines have the potential to reach over half of the Australian population through organised sport.^1^ In total, nine guidelines were articulated by a committee of experts and based on extensive literature review of existing mental health guidelines^22^ and mental health interventions in sport,^23^ a Delphi expert consensus study^24^ and key stakeholder input. The nine guidelines covered the general topics of: mental health literacy training; mental health support pathways and processes; responding to mental health crises; responding to major events that may impact mental health; having a mental health plan in place; reducing stigmatising attitudes; appointing a dedicated mental health officer; coach education and promoting well-being within the organisation. When implemented, the guidelines are expected to enable sports organisations to support the mental health and well-being of all participants. Given more than half of Australians aged 15+ years participate in or are associated with organised sport each year, and half of children aged 0–14 years participate in sport weekly (outside of school),^32^ the guidelines could have a significant impact on the mental health of a large segment of the population.

### Clinical and policy implications

The extent of the expected impact of the guidelines will be dependent on the rates at which community sports organisations implement the guidelines. Initial surveillance data regarding the current implementation strategies of sports organisations to promote mental health are needed. As with physical activity guidelines, one of the major benefits of having mental health guidelines is to enable the monitoring of compliance with the guidelines. Such monitoring can provide important data that will enable key areas of action nationwide to promote positive mental health and well-being in community sport. Ongoing monitoring will allow an understanding of the changes in rates of uptake of the guidelines over time—a key indicator of their success.

The extent to which the guidelines will have a meaningful impact is the extent to which they complement existing policies. For example, the Australian Sports Commission developed a suite of policies related to integrity, inclusivity and child safeguarding in sport.^33^,^35^ To the best of our knowledge, with some exceptions, the major policy omissions are those pertaining specifically to mental health and well-being. As such, national mental health guidelines for sport can complement and extend on the current suite of policies at the highest level of sport governance in Australia. Specifically, the guidelines respond to the need to provide a safe, trustworthy and fair sport sector, one of the four key priority areas identified in the Sport 2030 report.^36^ They can also enhance the effectiveness of mental health initiatives by national sports organisations and government bodies in Australia by providing an overarching national approach, and thereby also extending guidance to those sports and geographies without any current guidance in place. For example, the current set of guidelines can extend those offered by the AFL Mental Fitness Charter, which includes ‘Five Commitments’ and a ‘quick win action plan’ for clubs.^11^ These actions include significant overlap with the guidelines articulated here, including, increasing mental health literacy; promoting well-being; responding to events that have an impact on mental health; promoting a culture of well-being; increasing mental fitness skills and reducing risks to mental health. The overlap provides some evidence of the feasibility and acceptability of the guidelines, as well as evidence of an appetite for a national approach to promoting and protecting mental health in community sport.

Of critical importance to the effectiveness and impact of the guidelines will be to maximise their reach, adoption, implementation and maintenance.^37^ There needs to be a realistic and practical approach to their implementation through providing adequate support and resources to clubs, as well as training to ensure that sports clubs can effectively integrate the guidelines into their day-to-day functioning without compromising their core activities. This will require a whole-of-system approach,^15^ including working with the mental health sector, government bodies and national sports organisations to endorse, advocate, oversee and provide resources for the uptake and implementation of the guidelines. For example, national sports organisations and governments have an opportunity to formalise these guidelines in the form of policies that can have meaningful impact at a societal and sport-system level. State sports associations and/or bodies can also advocate, oversee and provide resources for the uptake and implementation of the guidelines, for example by providing coach education at a state or regional level, or by establishing relationships between a group of geographically limited sports organisations and mental healthcare providers. Finally, community sport organisations can take up the guidelines through the committed action of their administrators and members. As such, all levels of the sport system have an opportunity to contribute to the protection and promotion of mental health and well-being in community sport in Australia through coordinated action. Such action is urgently needed and can have a meaningful effect on mental health and well-being of a generation of Australians and contribute to the prevention of over 1.8 million cases of mental health problems.^38^

### Limitations and future directions

This study is not without its limitations. First, the primary challenge for the guidelines will be the extent to which community sport organisations have the time, resources and expertise to implement them in practice. While we have used a robust stakeholder codesign process and taken all care to articulate guidelines that are feasible, community sports organisations are inevitably under-resourced, undertrained and lack the mental health literacy and implementation knowledge to put in place their own strategies to protect and promote mental health.^39 40^ Recent research has suggested that sport clubs have differing levels of readiness to support mental health protection and promotion,^19^ and expecting sports clubs to protect and promote mental health while grappling these other issues adds an additional layer of complexity. To address these issues, the guidelines represent only the minimally acceptable standards for sporting clubs and have been designed to be flexible enough to be adapted to most sport contexts, ultimately minimising both the knowledge and resource requirements of each club.^16^ Similarly, the guidelines have been designed to ensure that the core purpose of community sport (ie, physical activity, having fun and making friends) is still incorporated throughout the guidelines and their implementation. Despite this, the guidelines would benefit from being disseminated with appropriate supporting material that outlines exactly how community sports organisations can best implement the guidelines, including recommended strategies, recommended programmes or providers and templates (see online supplemental material). There may also be a need for financial or human resource support for community sport organisations to implement the guidelines. Naturalistic case studies of the guidelines with Australian sport clubs are currently underway. The piloting of the guidelines in a naturalistic environment is imperative to both understand and ensure the adoption, implementation and maintenance of the guidelines by the end-users. It also provides an additional step to make any necessary changes to the guidelines and/or supporting material before engaging in a larger dissemination strategy. Second, as the guidelines have been developed with, and designed for, Australian community sport organisations, they may not be appropriate for other community sport environments outside of Australia. As such, future research should consider adapting the guidelines to be suitable to international contexts to ensure even greater reach and impact. Finally, the community sport organisations that participated in phase I and the follow-up focus groups may not be entirely representative of the Australia community sport sector. Despite our best efforts to purposely sample from a range of Australia sports clubs, it is likely that the clubs that did participate have an invested interest in mental health and well-being. Clubs that perhaps are not as knowledgeable or invested may not have participated, meaning there could be aspects of the guidelines that have been missed. However, future amendments and updates to the guidelines in the coming years may overcome this limitation.

## Conclusion

We have presented a set of nine evidence-based and actionable mental health guidelines for organised community sport in Australia. These guidelines address gaps in existing government policies and extend initial efforts from within the sport sector to articulate actionable, mental health promotion strategies for community sports organisations. When implemented, the expected benefit of the guidelines are to protect and promote mental health and well-being in community sport. Given the scale of preventable mental health problems in Australia,^6^ advocacy and the provision of resources to enable uptake an implementation of the guidelines are likely to be a cost-effective endeavour. There is an urgent need to identify the advocacy, policy and implementation initiatives that are required to ensure that the guidelines can maximise their reach, effectiveness, adoption, implementation and maintenance.

## Supplementary material

10.1136/bjsports-2024-108749 online supplemental file 1

## Data Availability

Data are available upon reasonable request.

## References

[1] Australian Sports Commission (2019). AusPlay survey results july 2018 - june 2019.

[2] Batista MB, Romanzini CLP, Barbosa CCL (2019). Participation in sports in childhood and adolescence and physical activity in adulthood: A systematic review. J Sports Sci.

[3] World Health Organization (2022). World Mental Health Report: Transforming Mental Health for All.

[4] Australian Bureau of Statistics (2021). Causes of Death, Australia.

[5] Australian Institute of Health and Welfare (2024). Prevalence and impact of mental illness.

[6] Australian Bureau of Statistics (2018). Mental Health.

[7] World Health Organization (2013). Comprehensive Mental Health Action Plan.

[8] Hoffmann MD, Barnes JD, Tremblay MS (2022). Associations between organized sport participation and mental health difficulties: Data from over 11,000 US children and adolescents. PLoS One.

[9] Eather N, Wade L, Pankowiak A (2023). The impact of sports participation on mental health and social outcomes in adults: a systematic review and the “Mental Health through Sport” conceptual model. Syst Rev.

[10] Vella SA, Swann C (2021). Time for mental healthcare guidelines for recreational sports: a call to action. Br J Sports Med.

[11] Hall K, Harris E, Couston N (2024). AFL mental fitness charter 2024-2027.

[12] SportWest Mental health and wellbeing community sport framework.

[13] Sport SA (2022). Mental fitness charter.

[14] VicHealth (2020). Mental health and wellbeing support in sport.

[15] Vella SA, Schweickle MJ, Sutcliffe J (2022). A Systems Theory of Mental Health in Recreational Sport. Int J Environ Res Public Health.

[16] Petersen JM, Drummond M, Elliott S (2024). Examining the promotion of mental health and wellbeing in Australian sports clubs. Sport Educ Soc.

[17] Petersen JM, Drummond M, Crossman S (2023). Mental health promotion in youth sporting clubs: predictors of stakeholder participation. BMC Public Health.

[18] Swierzy P, Wicker P, Breuer C (2018). The impact of organizational capacity on voluntary engagement in sports clubs: A multi-level analysis. *Sport Management Review*.

[19] Elliott S, Petersen J, Drummond M (2024). What are the perceived barriers for building and maintaining a culture of mental health support in Australian competitive youth sport?. J Appl Sport Psychol.

[20] National Health and Medical Research Council (2016). Guidelines for Guidelines.

[21] Liddelow C, Schweickle MJ, Sutcliffe JT (2022). Protocol for national mental health guidelines for community sport in Australia. BMJ Open Sport Exerc Med.

[22] Vella SA, Schweickle MJ, Sutcliffe JT (2021). A systematic review and meta-synthesis of mental health position statements in sport: Scope, quality and future directions. Psychol Sport Exerc.

[23] Sutcliffe JT, Graupensperger S, Schweickle MJ (2024). Mental health interventions in non-elite sport: a systematic review and meta-analysis. Int Rev Sport Exerc Psychol.

[24] Liddelow C, Schweickle MJ, Sutcliffe JT (2023). Defining the scope and content of mental health guidelines for community sport in Australia: A Delphi study. Psychol Sport Exerc.

[25] Minkler M, Wallerstein N, Minkler M, Wallerstein N (2003). Community-Based participatory research for health.

[26] Braun V, Clarke V (2013). Successful qualitative research: a practical guide for beginners.

[27] Braun V, Clarke V (2019). Reflecting on reflexive thematic analysis. *Qualitative Research in Sport, Exercise and Health*.

[28] Carter P, O’Donoghue KJM, Dworzynski K (2021). A demonstration of using formal consensus methods within guideline development; a case study. BMC Med Res Methodol.

[29] Jorm AF, Korten AE, Jacomb PA (1997). “Mental health literacy”: a survey of the public’s ability to recognise mental disorders and their beliefs about the effectiveness of treatment. Med J Aust.

[30] Sport Integrity Australia (2024). National integrity framework: member protection policy. https://www.sportintegrity.gov.au/sites/default/files/SIA225-0823-6-NIF%20Member%20Protection%20Policy-A-5%20%5BDIGITAL%5D.pdf.

[31] Good sports (2025). About good sports. https://goodsports.com.au/about-good-sports/.

[32] Australian Sports Commission (2023). Ausplay: national sport and physical activity report: October 2023. https://www.clearinghouseforsportclearinghouseforsportclearinghouseforsportclearinghouseforsport.gov.au/__data/assets/pdf_file/0004/1122754/AusPlay-National-Sport-and-Physical-Activity-Participation-Report-October-2023.pdf.

[33] Australian sports commission (2025). Integrity policies and programs. https://www.sportaus.gov.au/integrity_in_sport/integrity-policies-and-programs.

[34] Australian sports commission (2025). Inclusive sport. https://www.sportaus.gov.au/integrity_in_sport/inclusive-sport.

[35] Australian sports commission (2025). Child safeguarding. https://www.sportaus.gov.au/integrity_in_sport/child_safeguarding.

[36] Department of health (2018). Sport 2030: national sport plan. https://www.sportaus.gov.au/__data/assets/pdf_file/0005/677894/Sport_2030_-_National_Sport_Plan_-_201.

[37] Shaw RB, Sweet SN, McBride CB (2019). Operationalizing the reach, effectiveness, adoption, implementation, maintenance (RE-AIM) framework to evaluate the collective impact of autonomous community programs that promote health and well-being. BMC Public Health.

[38] Grummitt L, Baldwin JR, Lafoa’i J (2024). Burden of Mental Disorders and Suicide Attributable to Childhood Maltreatment. *JAMA Psychiatry*.

[39] Ferguson HL, Swann C, Liddle SK (2019). Investigating Youth Sports Coaches’ Perceptions of Their Role in Adolescent Mental Health. J Appl Sport Psychol.

[40] Finch CF, Donaldson A (2010). A sports setting matrix for understanding the implementation context for community sport. Br J Sports Med.

