# Supplementary material for: Development of Australian mental health guidelines for community sport
Source: Br J Sports Med. 2025 Feb 19;59(9):e108749. doi: 10.1136/bjsports-2024-108749 (PMC12171466; doi:10.1136/bjsports-2024-108749)
Supplement: online supplemental file 1 [file bjsports-59-9-s001.pdf]

MENTAL HEALTH GUIDELINES

FOR COMMUNITY SPORT IN AUSTRALIA

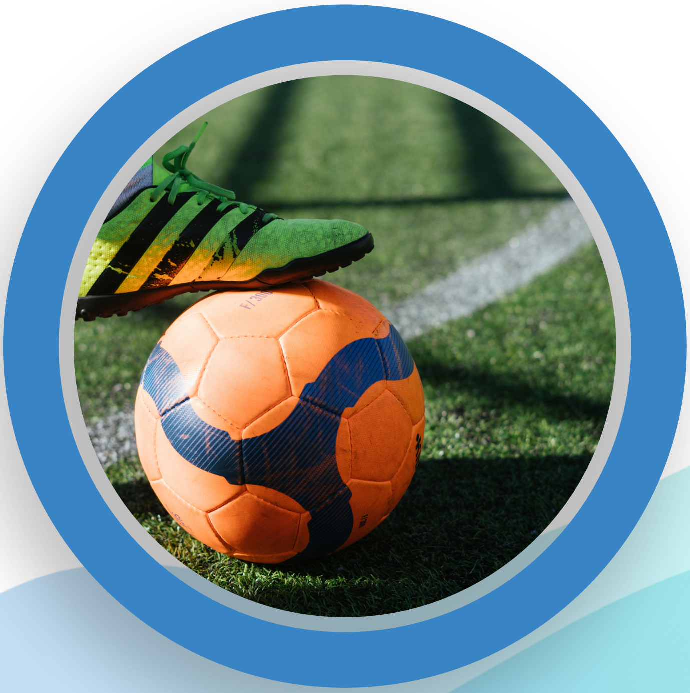

UNIVERSITY OF WOLLONGONG AUSTRALIA

MENTAL HEALTH GUIDELINES FOR COMMUNITY SPORT

CONTENTS

1. GLOSSARY OF TERMS PAGE 1
2. INTRODUCTION PAGE 2
3. GUIDELINE DEVELOPMENT PAGE 4
4. THE GUIDELINES PAGE 5
   4.1 PROMOTING WELLBEING PAGE 6
   4.2 REDUCING MENTAL HEALTH STIGMA PAGE 9
   4.3 PROMOTING COACH DEVELOPMENT PAGE 13
   4.4 INCREASING MENTAL HEALTH LITERACY PAGE 17
   4.5 LOCATING APPROPRIATE HELP PAGE 21
   4.6 RESPONDING TO A MENTAL HEALTH EMERGENCY PAGE 25
   4.7 RESPONDING TO MAJOR EVENTS THAT MAY IMPACT MENTAL HEALTH PAGE 28
   4.8 IMPLEMENTING A MENTAL HEALTH CHAMPION PAGE 30
   4.9 DEVELOPING A MENTAL HEALTH ACTION PLAN PAGE 34
5. REFERENCES PAGE 36
6. RESOURCES AND TEMPLATES PAGE 38
   MENTAL HEALTH ACTION PLAN
   MENTAL HEALTH EMERGENCY PLAN
   MAJOR EVENTS RESPONSE PLAN

1. GLOSSARY OF KEY TERMS

**Mental Health Literacy**	having the knowledge and beliefs about mental disorders which aid their recognition, management or prevention
**Mental Health Disorder**	a clinically significant disturbance in an individual's cognition, emotional regulation, or behaviour
**Mental Health Stigma**	the negative stereotypes about people living with mental health problems
**Mental Health Champion**	a nominated individual within an organisation that is the expert in everything related to mental health and wellbeing
**Mental Health Emergency or Crisis**	any immediate event or situation in which an individual's behaviour/s puts them at risk of hurting themselves or others
**Bullying**	a form of harassment, either verbal and physical

2. INTRODUCTION

The mental health and wellbeing of all people involved in sport is imperative. These mental health guidelines have been developed in conjunction with community stakeholders and Australian experts in the field of sport, mental health and sport psychology. The guidelines have been designed to support sports club in both promoting positive mental health and wellbeing and responding to poor mental health, with a focus on being proactive rather than reactive. We believe that a psychologically safe sport club will implement, or already have in place, all of the below guidelines. This will mean that not only individual members of the club, but also the larger club and community may benefit positively.

Nine guidelines for promoting and protecting the mental health and wellbeing of sport participants and members have been developed. The guidelines represent the minimum acceptable standards that a sport organisation should be doing to protect its members from psychological harm.

The guidelines are:

- recommendations for best practice, not policy, to allow individual sport clubs and associations some flexibility on how they are implemented
- recommended for use in all levels of community sport (team, club, association), with all age groups, genders, and cultures
- should be read and actioned by the committee, but have been developed to ensure positive mental health benefits to all sport members, including players, coaches, officials, and committee members
- designed to complement and be applied along with other current governing guidelines and legislation in sport, or other campaigns:
    - Member Protection Policy
    - Inclusion Policy
    - Good Sports (drug and alcohol in sport)
    - R U OK? Day
- effective when implemented alongside other guidelines and policies

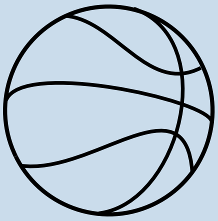

2. INTRODUCTION

It may be that your sport or club has already implemented one or more of these guidelines to some capacity. However, we believe for the full benefits to be achieved, all guidelines should be addressed or implemented over the course of the next few years.

There is no specific order in which the guidelines should be implemented, but some guidelines will naturally need to occur before another can be successfully implemented. Implementing the guidelines to their full extent will take some initial groundwork. However, they have been designed in a way to ensure that once they are implemented, they will only need to be altered or improved occasionally.

This document sets out:

- recommendations for guideline implementation, depending on the sport and club type.
- specific recommendations for who, how, and when each guideline can/should be implemented

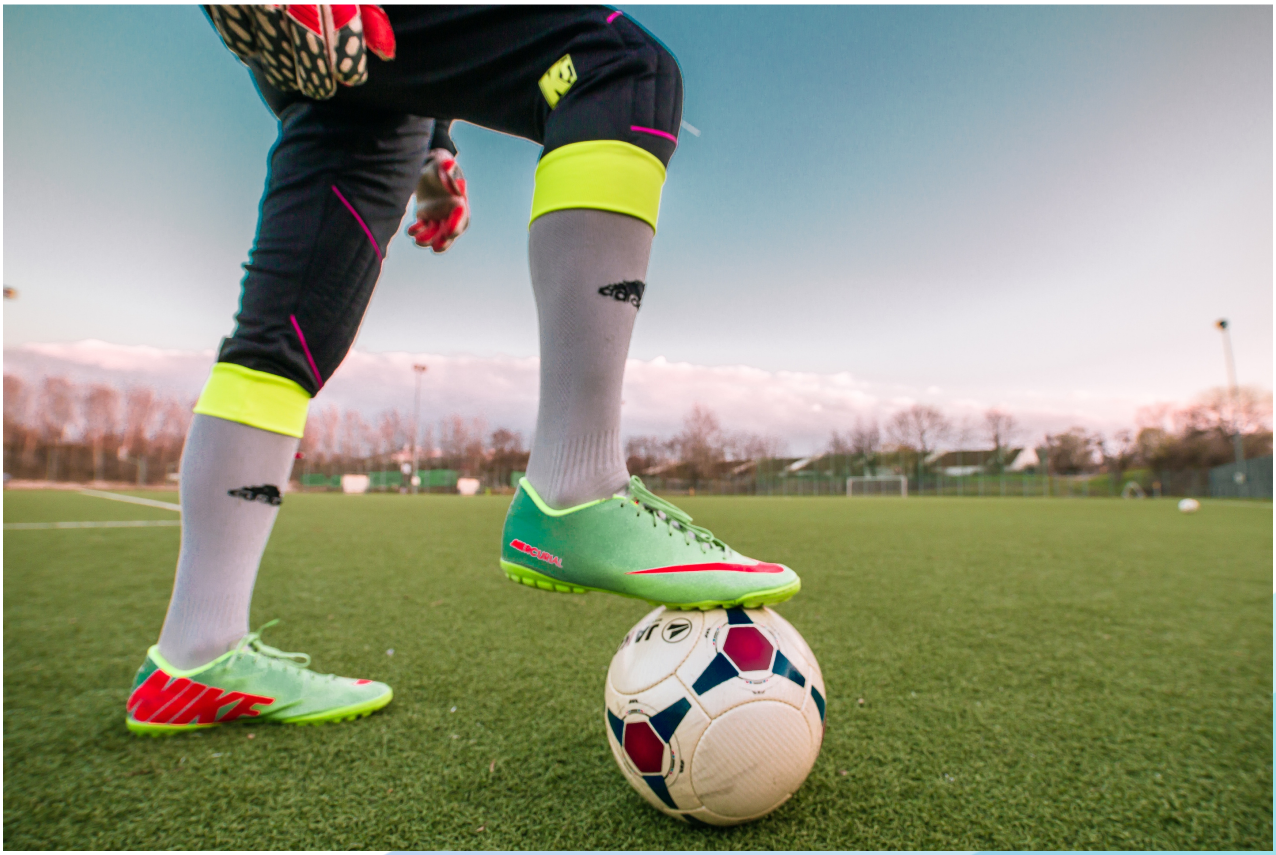

3. GUIDELINE DEVELOPMENT

This project is a government-funded project aimed at developing and implementing feasible and acceptable guidelines for community sport in Australia. Funded by the Australian Research Council (ARC) in 2020, the development of the guidelines has been ongoing, ensuring collaboration with both members of community sport clubs and experts in sport and mental health from around the country.

In May 2021, an expert panel was created to assist with the guideline development. The experts on this panel were sourced from universities, Government and not-for-profit organisations around Australia. All members that were invited were deemed to be an expert in the area of mental health and sport, or similar.

In August 2021, a study was conducted with the 21 members of the expert panel to establish a set of key priority areas and recommendations on the purpose and scope of future guidelines.

In September-November 2021, members from community sport clubs around Australia participated in a series of online focus groups. Coaches, committee members, parents and players shared their preferences and opinions for what future mental health guidelines for sport should focus on.

In February 2022, all members of the expert panel were invited to Wollongong for a three day National Consensus Meeting. This meeting focussed on developing and drafting the guidelines, based on the findings of the two studies above, and other research in the area.

In September-October 2022, sport club members that participated in the previous focus groups were re-contacted and invited to review the draft guidelines. These focus groups focussed on understanding the usability and acceptability of the guidelines. Any feedback was then incorporated, with the final result being this document.

4. THE GUIDELINES

Promoting Wellbeing: Community sport clubs need to demonstrate a commitment to enhancing the wellbeing of everybody involved in the club.

Reducing Mental Health Stigma: Community sport organisations demonstrate an ongoing commitment to promoting positive attitudes about mental health.

Promoting Coach Development: Community sport organisations need to support their coaches to participate in ongoing coach development in methods that promote safe/supportive coaching.

Increasing Mental Health Literacy: Community sport clubs need to demonstrate a commitment to enhancing the mental health literacy of their key stakeholders.

Locating Appropriate Help: Clubs need to demonstrate an awareness on what appropriate mental health services are available, and how to access them.

Responding to a Mental Health Emergency: Community sport organisations have a plan for how to respond to an identified mental health emergency (e.g., risk or suicide or harm to self/others).

Responding to Major Events that May Impact Mental Health: Community sport organisations have a plan for how to respond to major events or critical incidents that may impact the psychological wellbeing of their members.

Implementing a Mental Health Champion: Sport clubs and organisations need to have a mental health champion who actively oversees and contributes to the implementation of the mental health guidelines.

Developing a Mental Health Action Plan: Community sport organisations have a completed up-to-date mental health action plan outlining how they will prevent, promote and respond to mental health and wellbeing in sport.

4.1 Promoting Wellbeing

Promoting Wellbeing: Community sport clubs need to demonstrate a commitment to enhancing the wellbeing of everybody involved in the club.

Wellbeing is more than the absence of a mental illness [16]. Wellbeing includes things like satisfaction with life, feeling happy and content, having good social relationships, and feeling like you make a positive contribution to society [16]. Wellbeing is critical to one's overall mental health and is also a great protector against mental health problems [17]. Sports clubs already make a positive contribution to the wellbeing of the people who are involved in them [18]. By simply doing what sports clubs do, and doing it well, every community sports club can enhance the wellbeing of everybody involved in the club.

WHO FOR?

Sports clubs should demonstrate a commitment to enhancing the wellbeing of everybody who is involved in the club - including players, coaches, committee members, parents, officials, and volunteers.

WHO IS RESPONSIBLE?

Each sport organisation committee needs to ensure that they have programs and/or processes in place to enhance wellbeing. This may be a role that is shared between the President and Vice-President, as well as the Mental Health Champion (see 4.8). These programs and/or processes may all form part of the Mental Health Action Plan (see 4.9) that is developed in conjunction with the nominated club Mental Health Champion (see 4.8).

HOW?

Being involved in a sports club is typically good for wellbeing [19]. Indeed, there are many ways in which sports club contribute to wellbeing. Simply by doing these things well in their usual running's of the club, clubs can enhance the wellbeing of everybody involved.

4.1 Promoting Wellbeing

Promoting Wellbeing: Community sport clubs need to demonstrate a commitment to enhancing the wellbeing of everybody involved in the club.

Some examples of ways to enhance wellbeing in your club include:

- Reducing dropout from the club/team/association or sport;
  - See 4.2 for more information
- Enhancing the quantity and quality of social interactions within the club;
  - Have a regular team or whole club social events
- Offering a place for people to spend time and interact - a place to belong;
  - If feasible, a clean and safe clubhouse is a great place for this
- Providing a psychologically safe sport environment (on and off the field) free from harassment, bullying and anti-social behaviour - see resources below for how to implement this
  - Tips for parents
  - Tips for committees
- Giving people at all levels an opportunity to contribute to the club;
  - Allow individual players or members to contribute to club decisions
- Promoting inclusivity - see resources below for how to implement this
  - How to be an inclusive club
  - Club inclusivity checklist
- Promoting high levels of physical activity within training and competition;
  - Skills training is important, but so is general physical activity and exercise
- Serving healthy and nutritious food within the club - see resources below for how to implement this
  - Food toolkit
- The responsible service of alcohol – see resources below for how to implement this
  - Alcohol management

Each of these measures and/or actions is reflective of a good quality sports club. Simply by being a good quality sports club, wellbeing can be enhanced.

This information will inform the *Promoting Wellbeing* section of the Mental Health Action Plan (see Guideline 4.9).

4.1 Promoting Wellbeing

Promoting Wellbeing: Community sport clubs need to demonstrate a commitment to enhancing the wellbeing of everybody involved in the club.

WHEN?

Enhancing wellbeing is an ongoing commitment and should be undertaken year-round. The committee and nominated Mental Health Champion should conduct biennial (every 2 years) reviews of their Promoting Wellbeing plan.

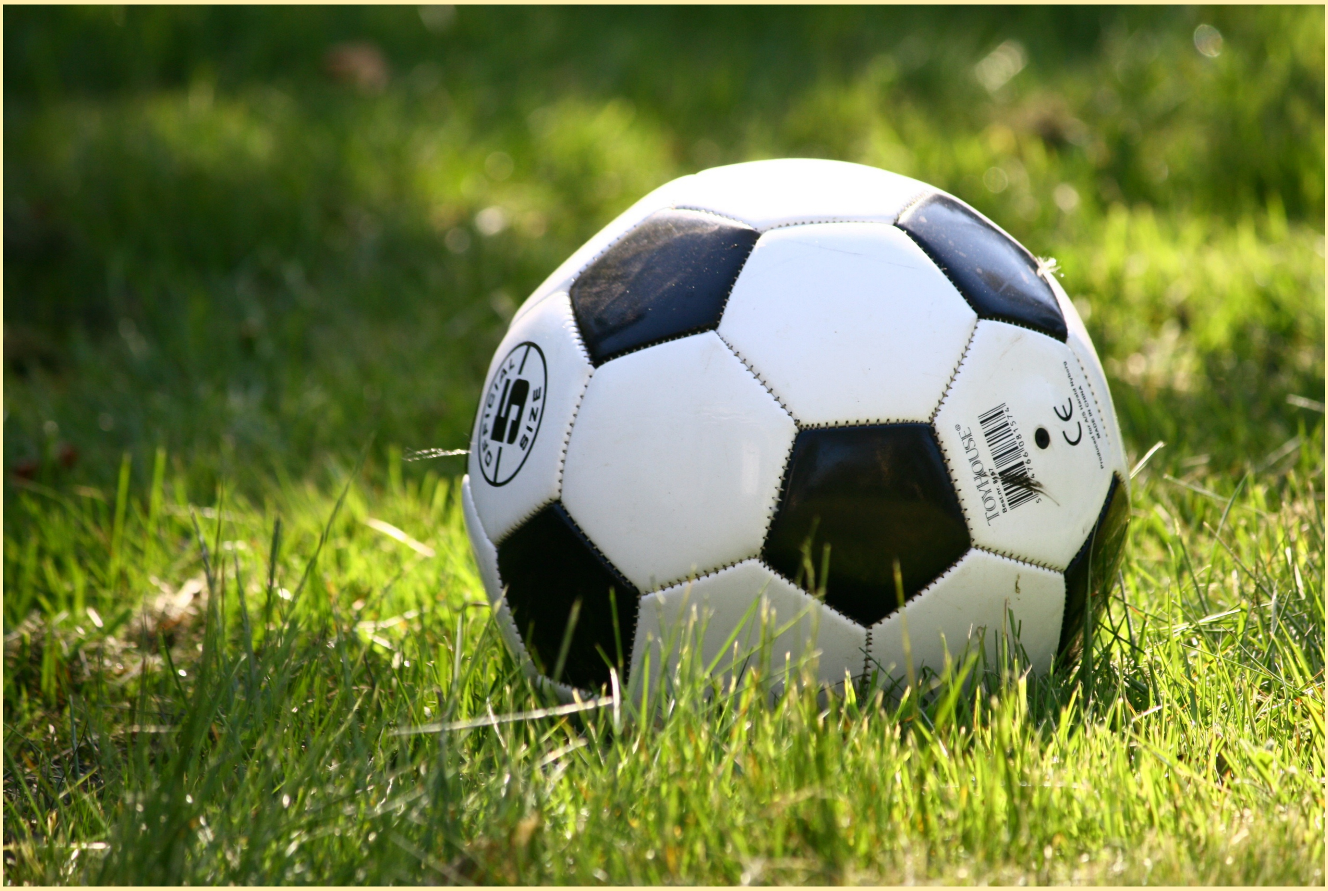

4.2 Reducing Mental Health Stigma

Reducing Mental Health Stigma: Community sport organisations demonstrate an ongoing commitment to promoting positive attitudes about mental health.

Many aspects of society, such as places, people, and cultures, can experience negative attitudes and stereotypes. This is often called 'stigma'. Mental health stigma is defined as the negative stereotypes about people living with mental health problems [5]. There are two common types of stigma related to mental health problems. The first is "public stigma" which refers to common stereotypes about mental health problems and individuals who experience these problems in society. The second type, "internalised stigma" or "self-stigma" refers to how an individual with a mental health problem absorbs public stigma and stereotypes as part of their identity [6]. When someone with a mental health problem experiences stigma, this can have negative impacts on their desire to seek help and thus can make the mental health problem worse [7].

It is important that community sport organisations assist in shifting these negative attitudes in day-to-day life, but also within their own sport organisation. Part of the solution to changing this is to promote positive attitudes towards mental health problems, such as through a change in language or behaviours towards people experiencing mental health difficulties. For example, by labelling someone as their illness (i.e., they are psychotic), rather than by experiencing an illness (i.e., someone experiencing psychosis), individuals can feel judged, unaccepted and worthless. So, by changing things such as how we describe people experiencing a mental health problem, we can shift the overall attitudes people have towards mental health problems.

In addition, by having positive attitudes towards mental health and people experiencing mental health issues, you are contributing to a mentally healthy and psychologically safe environment for your members. By engaging in sport and physical activity, people already experience positive benefits for their mental health and wellbeing. So, by making a few additional changes, these positive benefits can be enhanced and allow for all members to receive the same benefits from participation. This guideline plays a role in ensuring Promoting Wellbeing (see 4.1) is being fulfilled.

4.2 Reducing Mental Health Stigma

Reducing Mental Health Stigma: Community sport organisations demonstrate an ongoing commitment to promoting positive attitudes about mental health.

WHO FOR?

It is important that all members of the club and associations, such as committee members, volunteers, coaches, officials, parents and players, are promoting positive attitudes towards mental health and wellbeing.

WHO IS RESPONSIBLE?

It is the responsibility of all members of the club and association to promote positive attitudes towards mental health. However, by having positive change at the top of the club (e.g., the committee putting in initiatives to educate members on positive language), this is more likely to trickle down to the other members in the club. This 'trickle-down effect' has been shown in other areas of society, such as in corporate organisations [8]. Therefore, it is up to the committee members and specifically the Mental Health Champion (see 4.8) to make the initial (and ongoing) positive changes to thus benefit the other members.

HOW?

There are many ways in which positive attitudes towards mental health can be fostered in a sport organisation. According to BeyondBlue, there are two main stigma-reduction approaches that can be taken:

1. **Educational approaches** – Providing information and resources which challenge inaccurate and untrue stereotypes, and instead replace them with correct information.
2. **Contact approaches** – This involves having direct interpersonal contact with someone with a mental health problem (e.g., friendship, relationship, team member).

At a minimum, sport organisations must ensure they are incorporating some of the below suggestions into the everyday management of the sport organisation. Each organisation should aim to do something more than what they are currently doing. To do more, clubs can incorporate some of the below recommendations into their constitution and club rules/policy.

4.2 Reducing Mental Health Stigma

Reducing Mental Health Stigma: Community sport organisations demonstrate an ongoing commitment to promoting positive attitudes about mental health.

Some specific ways to reduce mental health stigma include, but are not limited to:

- Having open conversations in the club about mental health and wellbeing
- Ensuring when discussing or educating others on mental health that accurate facts are used (referring to information and resources is key here)
    - Can supply fact sheets or pamphlets from appropriate resources on the club website
- Being conscious of stigmatising language and pointing out to others how they can improve their use of language.
    - This website may assist you and your club in understanding what to say and what not to say about mental health problems
- Educating the members of the sport organisation using evidence-based programs and workshops (see 4.4 and 4.5 for further direction)
- Notice and appropriately respond to individuals who may be struggling (see 4.5)
- Treating mental health problems equally to physical injuries/illnesses (e.g., recovery time off)
- Raise money for mental health charities as a club or organisation (e.g., Lifeline, BeyondBlue, Black Dog Institute)
    - Have a mental health day or week to focus on mental health and wellbeing activities within the organisation
- Inviting individuals with a lived-experience of a mental health problem, and who have mental health training, to safely share their story
- Articulate mental health and wellbeing as a priority of the club/sport organisation
- Integrate mental health into club/sport organisation values/mission/philosophy

If you wish to read more about the importance of reducing stigma, and ways this can be done in your sport organisation, please visit BeyondBlue.

4.2 Reducing Mental Health Stigma

Reducing Mental Health Stigma: Community sport organisations demonstrate an ongoing commitment to promoting positive attitudes about mental health.

WHEN?

As part of your club's Mental Health Action Plan (see 4.9), we recommend including some, or all, of the above recommendations in ways to promote and foster positive attitudes towards mental health in your club. Clubs may wish to re-assess or change their initiatives when they update their Mental Health Action Plan (see 4.9). However, it is important to ensure that reducing mental health stigma is an ongoing commitment within the club.

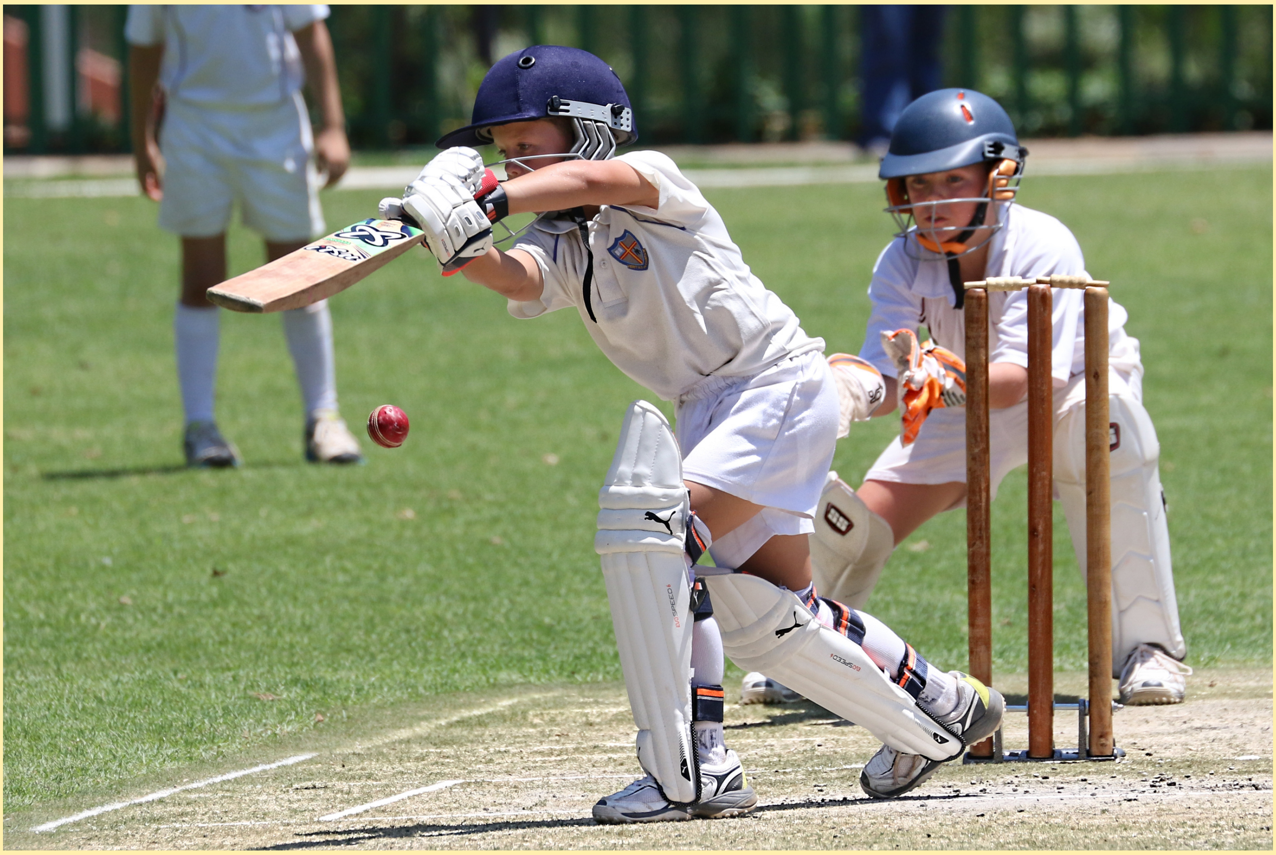

4.3 Promoting Coach Development

Promoting Coach Development: Community sport organisations need to support their coaches to participate in ongoing coach development in methods that promote safe/supportive coaching.

Recreational sports coaches can meaningfully influence the psychological development of sport participants. Because of this, the provision of a psychologically safe and motivating environment are key components of effective coaching [9,10]. This is reflected in coaches' preferences for coach education, where popular topics include motivation and supportive approaches to coaching [11]. However, while some coaching resources are available for coaches in regard to mental health and wellbeing [12], formal coach education courses are unlikely to prepare coaches for creating psychologically safe, supportive, and motivating environments. Nonetheless, there are evidence-based approaches to coaching available that can help coaches to coach in ways that are psychologically safe and supportive [13]. Whilst different coaching courses are appropriate for different levels of sport coaches (e.g., recreational, or high-performance sport), it is important that any approach to coaching is athlete-centred and to the benefit of the players.

It is important that coaches are appropriately trained in how to coach safely and effectively, on top of already being up-to-date and trained in sport organisation specific courses such as child and member protection.

WHO FOR

All coaches/managers should be supported by the committee members to participate in ongoing coach development in methods that promote safe/supportive coaching.

WHO IS RESPONSIBLE

It is each club's responsibility to ensure that all coaches are supported to participate in ongoing coach development in methods that promote safe/supportive coaching. In particular, it is up to the members of the committee to take responsibility for organising coach education and training, or at a minimum, monitoring coach engagement in new coaching courses. This process should also be overseen by the Mental Health Champion (see Guideline 4.8).

4.3 Promoting Coach Development

Promoting Coach Development: Community sport organisations need to support their coaches to participate in ongoing coach development in methods that promote safe/supportive coaching.

In addition, sport associations and governing bodies should be responsible for ensuring coaches within their sport clubs and teams are participating in coaching development opportunities related to mental health and wellbeing. Associations may also consider attendance and participation in such education and training as a prerequisite for coaching in their region.

HOW

Evidence-based coach training is effective and should be the first option where feasible. Some accredited coaching courses may already include content and skills related to psychologically safe coaching. However, often, this is not enough. It is also important that coaches do not repeat the same coaching course each year, but rather engage in professional development every few years by completing new training courses.

There are a number of coach education programs that are available at a range of costs. It is important, however, that all current and future coaches of sport are certified to coach based on the requirements of the sport and/or level. Some programs for coach development that we recommended are:

Essential Skills Course: This free online general coaching skills course from Sport Australia guides coaches through eight modules to help them create fun, safe and inclusive environments. This course offers sport specific content (athletics, netball, swimming, squash) as well as more general content for all sport types.

Tackle Your Feelings: AFL-specific program for coaches focussed on teaching AFL coaches skills for ensuring a psychologically safe environment.

Player-Centred Coaching Course: The free online course developed by Deakin University will give current and future coaches an introduction to player-centred coaching principles.

4.3 Promoting Coach Development

Promoting Coach Development: Community sport organisations need to support their coaches to participate in ongoing coach development in methods that promote safe/supportive coaching.

Inclusive Coaching: Sport Integrity Australia in collaboration with Play by the Rules have developed a free online course to teach community sport coaches approaches and strategies for ensuring an inclusive environment for all.

Let Kids be Kids: This free online short course developed by Sport Integrity Australia in collaboration with Play by the Rules was developed to understand and address poor behaviours on the sideline. This course has a particular focus on junior sport and the actions of spectators, and how to reduce this behaviour.

Wellbeing Toolkit for High Performance Sport Coaches: This course developed by the Australian Institute of Sport is aimed at coaches involved in coaching in high performance/elite sport and associated pathways. This course provides coaches with three modules to provide them with practical, evidence-based tools to improve their personal wellbeing and capacity, as well as the wellbeing of their athletes.

If programs such as the above are not accessible or feasible, other evidence-based approaches to coaching that can help coaches to promote safe and supportive environments include:

- Transformational approach to coaching (e.g., athlete-centred coaching)
- Autonomy-supportive coaching
- Mastery approach to coaching

Each of these programs could be delivered by an appropriately qualified sport psychology practitioner.

For a directory of sport psychology practitioners, you can visit Find a Psychologist | APS (psychology.org.au) and select "Sport and Exercise Psychology" under the area of practice. Visiting a sport psychologist will have out-of-pocket costs. These costs will slightly vary between practitioners, practices, and regions.

4.3 Promoting Coach Development

Promoting Coach Development: Community sport organisations need to support their coaches to participate in ongoing coach development in methods that promote safe/supportive coaching.

To ensure coaches in your sport club or association are supported in their development as a coach, we also recommend sport organisations change their coaches code of conduct to align with this changing trend in coaching. This is the new 'best practice' of sport coaching.

WHEN

All current and future sport coaches must be certified coaches in their respective sport. Ideally, all coaches will undertake additional evidence-based coach development during their role as a coach, or if feasible, prior to coaching. It is important that training and development is continuous, so ensuring sport organisations offer training of this nature every 3 years is imperative.

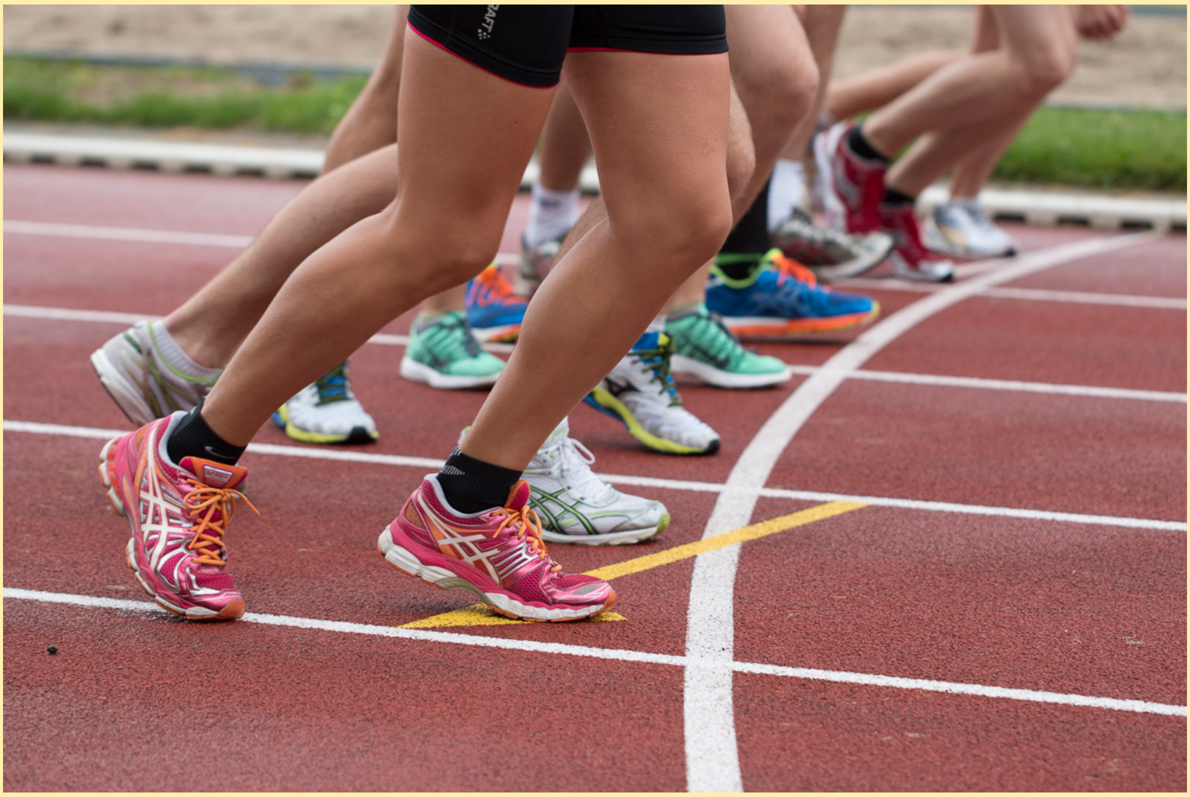

4.4 Increasing Mental Health Literacy

Increasing Mental Health Literacy: Community sport clubs need to demonstrate a commitment to enhancing the mental health literacy of their key stakeholders.

Mental health literacy refers to having the "knowledge and beliefs about mental disorders which aid their recognition, management or prevention"[1]. More specifically, it includes:

- the ability to recognise specific mental health issues
- knowing how to seek the correct mental health-related information
- knowing the risk factors and potential causes of mental health disorders
- knowledge of the different types of self-treatment
- knowledge of the professional support available
- having the right attitudes to promote the recognition of mental health disorders and seeking the appropriate help

Evidence suggests that mental health literacy training is beneficial for many members of sport clubs, such as the players themselves, parents, and coaches. In three large Australian studies, mental health literacy training was found to increase adolescents, parents, and coaches depression and anxiety literacy (i.e., their understanding of these common mental health disorders), intentions to seek help from formal sources such as GP's or psychologists, confidence to seek mental health information, confidence to assist someone experiencing a mental health disorder, more positive attitudes towards people with a mental health disorder, resilience, and also decreased emotional distress [2–4]

High levels of mental health literacy in your club would mean that members would also likely experience increases and improvements in their knowledge and understanding of mental health disorders, attitudes towards people experiencing mental health disorders, intentions and confidence to seek appropriate help and perhaps experience lower levels of emotional distress. This, overall, would not only assist your members in their day-to-day interactions within the club, but also in their everyday life.

4.4 Increasing Mental Health Literacy

Increasing Mental Health Literacy: Community sport clubs need to demonstrate a commitment to enhancing the mental health literacy of their key stakeholders.

WHO FOR

Whilst everyone in sport has a role to play in enhancing mental wellbeing in sport, at the very least, a commitment to enhancing the mental health literacy of all club committee members (e.g., President, Secretary, Development Officers) should be demonstrated.

Coaches, managers, team captains and other individuals in leadership roles have the most direct contact with players. Therefore, it is highly recommended that these members also receive mental health literacy training. This training should be in addition to the training recommended for coaches as part of 4.3 Promoting Coach Development.

To do more, clubs can offer training and/or resources to players, parents, officials and/or volunteers.

WHO IS RESPONSIBLE

Each club or team committee is responsible for ensuring the relevant members are committing to being mental health literate. It is the responsibility of the committee-appointed Mental Health Champion (see 4.8) to ensure action is being taken.

Regional associations or sport bodies may also monitor compliance with this guideline through annual or biennial reporting of engagement in one or more of the below evidence-based training programs.

Head sport bodies, such as state and national bodies need to ensure they are promoting the importance of sport and committee members being mental health literate.

4.4 Increasing Mental Health Literacy

Increasing Mental Health Literacy: Community sport clubs need to demonstrate a commitment to enhancing the mental health literacy of their key stakeholders.

HOW

Evidence-based mental health literacy training is effective and should be the first option where feasible. Evidence-based means there is scientific research and evidence to show that a program is effective in teaching and improving mental health literacy. If a program does not have evidence to show it is effective, it may do more harm than good. For example, it may make players or coaches feel distressed or deter them from seeking help.

There are several sport-specific mental health literacy programs that are available at a range of costs and targeted at different groups in sport. Some programs last only a few hours and others may require a few days. It is important that the appropriate program/s are selected for each target group. Some programs that we recommended, but are not limited to, include:

Sport-Specific Programs

- **Ahead of the Game**: Sport-specific programs available for players, coaches, and parents. Ahead of the Game has recently partnered with the AFL.
- **Tackle Your Feelings**: AFL-specific program for coaches, committee members and support staff.
- **Read the Play**: Sport-specific program aimed at young Australians in junior sport.

General Programs

- **Mental Health First-Aid Training**: General mental health training for people of all ages.
- **LivingWorks Suicide Prevention Training:** General suicide prevention training for organisations and workplaces.

It is important to note that programs that are not in this list are unlikely to currently be evidence-based, and therefore their effectiveness and the likelihood of causing harm is unknown.

4.4 Increasing Mental Health Literacy

Increasing Mental Health Literacy: Community sport clubs need to demonstrate a commitment to enhancing the mental health literacy of their key stakeholders.

WHEN

All current committee members and coaches should receive mental health literacy training as soon as possible. It should also be a priority that any new committee members are provided the opportunity to receive training within the first 3 months of their position. Training should be updated in-line with the recommendations from the mental health literacy program (e.g., mental health first-aid training requires a refresher course every three years), or biennially (every two years).

For all other club members (e.g., players, officials, volunteers), we recommend providing biennial education to ensure all new members of the sport are up to date.

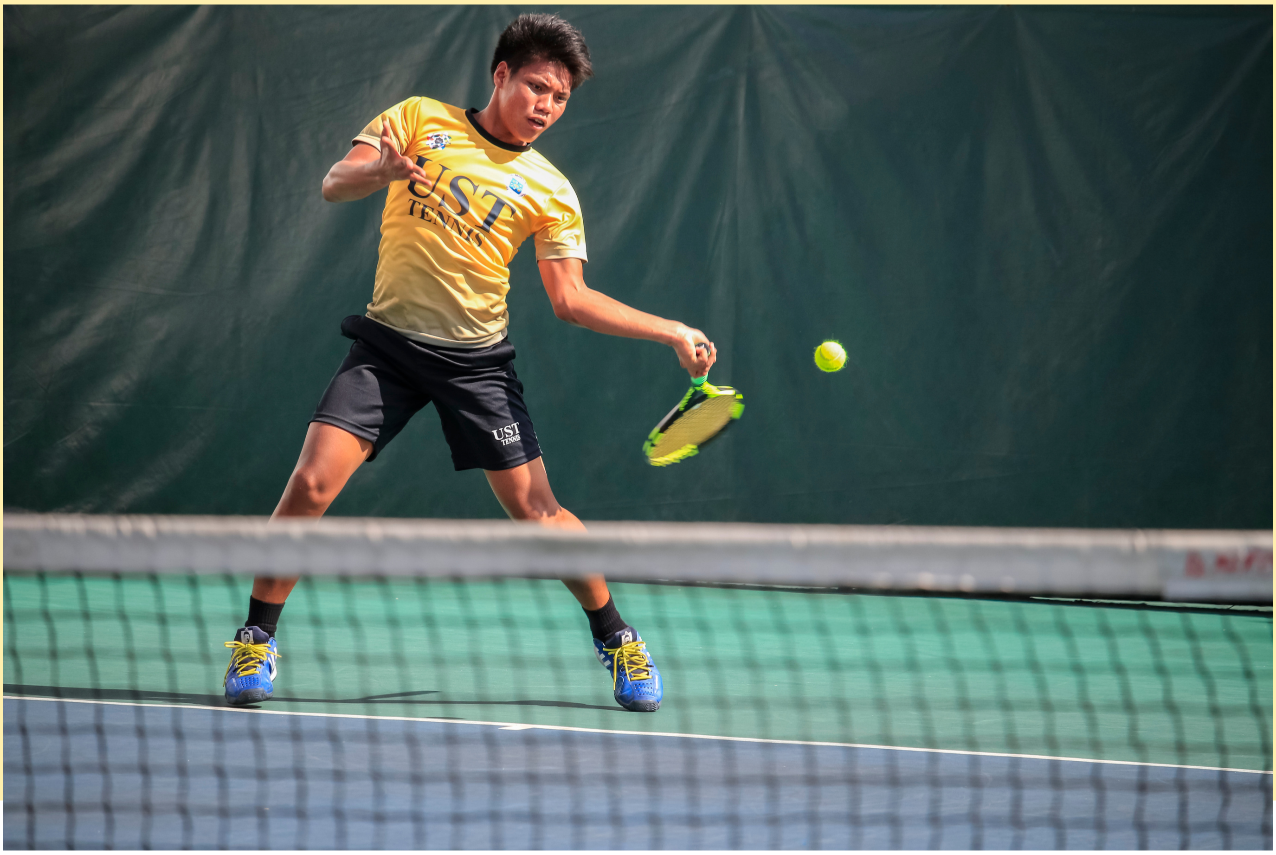

4.5 Locating Appropriate Help

Locating Appropriate Help: Clubs need to demonstrate an awareness on what appropriate mental health services are available, and how to access them.

Mental health services refer to online or in-person locations where individuals can seek assistance for their mental health and wellbeing. Having a list of suitable services available is important to ensure if a club member needs help with their mental health, they know where and how they can access specific, appropriately qualified evidence-based services. Mental health services can save lives and improve the quality of life of people experiencing mental health problems. It is also important to have this information, so club members know where to seek help in a mental health emergency (see 4.6) or a major event affecting mental health (see 4.7).

WHO FOR

At the very least, an awareness of appropriate mental health services is required by committee members, and coaches/managers. It is also important that the Mental Health Campion (see Guideline 8) is up-to-date with the current list of appropriate mental health services and can recommend them to club members when necessary.

If clubs would like to do more, other club members (e.g., players, parents, volunteers) could also be aware of this list and where to locate it.

WHO IS RESPONSIBLE

It is the responsibility of the Mental Health Champion (see 4.8) to work with the committee members in putting together this resource and ensuring it is easily accessible when in need.

Local and regional associations and sport bodies also have a responsibility in facilitating this within their district. Sport bodies need to ensure committees and clubs are actively aware of appropriate mental health services.

4.5 Locating Appropriate Help

Locating Appropriate Help: Clubs need to demonstrate an awareness on what appropriate mental health services are available, and how to access them.

HOW

Clubs should incorporate a list of appropriate mental health services in their Mental Health Action Plan (see Guideline 9). This list should contain the name, location and contact details of each service. This list of resources and services should not only be listed in your club Mental Health Action Plan, but also easily accessible and available in the club house and the website. This will mean all members of the club/sport will have access to the list.

It is important that service providers are established, and the individual practitioner is registered through the Australian Health Practitioner Regulation Agency (AHPRA). Some specific services that we recommend, but are not limited to, include:

Head to Health (online): Provided by the Australian Government, Head to Health is an online platform that can help you find digital mental health services and resources from around Australia. It brings together apps, online programs online forums, phone services and a range of other digital resources to assist anyone with their mental health and wellbeing. Head to Health is a good starting point for identifying suitable online services for a range of mental health concerns.

BeyondBlue (online): An online service and website that provides resources on mental health and wellbeing, as well as ways to locate appropriate support. They also offer an online, immediate support program for individuals that may need some assistance with mental health concerns.

Headspace (online and in-person): Headspace is a mental health foundation and service specifically for young people. They offer both online and in-person professional support, as well as employment assistance and support. It is likely there is a Headspace centre near you which also offers in-person psychological support.

4.5 Locating Appropriate Help

Locating Appropriate Help: Clubs need to demonstrate an awareness on what appropriate mental health services are available, and how to access them.

Lifeline (online): Lifeline Australia is a free online and phone suicide support service. They are a national charity that provides 24/7 crisis support and suicide prevention services to Australians of all ages. Lifeline can be contacted through their website or over the phone, by dialling 13 11 14. Don't like talking over the phone? They also offer a text messaging service.

Kids Helpline (online): Kids Helpline is an online and phone counselling service for kids and adolescents, as well as parents. Kids Helpline also have a range of digital resources and information for navigating mental health and wellbeing. No problem is too big or small for Kids Helpline and they can be contacted 24/7 on 1800 55 1800 or via email or webchat on their website.

Mindspot (online): Mindspot provides free digital mental health services to adults around Australia for a range of mild-moderate mental health concerns. Mindspot offers free mental health assessments and treatment from trained mental health professionals.

If clubs would like to do more, they can establish a relationship with a local, registered mental health service provider (e.g., local Headspace), if there is one in their locality.

If a club is unsure what appropriate mental health services are available in their region, contacting the state sport body or regional association for some initial contacts is a great place to start. It is possible that some districts and regions already have existing partnerships with some mental health service providers.

4.5 Locating Appropriate Help

Locating Appropriate Help: Clubs need to demonstrate an awareness on what appropriate mental health services are available, and how to access them.

Types of mental health providers that you may come across include:

- **Psychologists**
  - Clinical psychologists are the experts in assessing and treating moderate-severe mental, emotional, and behavioural disorders.
  - Generally registered psychologists are appropriate for mild-moderate mental health concerns, as well as a range of other psychological and behavioural concerns.
  - Sport psychologists are also trained in the assessment and treatment of mild-moderate mental health concerns but tend to work with those involved in sport. Sport psychologists can also assist in psychological functioning related to performance.
  - Find an appropriate psychologist near you, here
- **Other mental health professionals**
  - School counsellor
  - Counsellor
  - General Practitioners (GPs) who can refer you to an expert
- **Other healthcare professionals who are trained in mental health care (a good addition to seeing a psychologist)**
  - Occupational therapists
  - Speech pathologists
  - Paediatricians
  - Psychiatrists
  - Social Workers

WHEN

It is important that this list of appropriate mental health services is incorporated into the Mental Health Action Plan (see 4.9) as soon as possible. All current and future committee members and coaches/managers should be knowledgeable of this list, where to access it and how to use it when needed. This list of appropriate services should be updated regularly, such as biennially, to ensure the contact details are correct and new services or providers are included.

4.6 Responding to a Mental Health Emergency

Responding to a Mental Health Emergency: Community sport organisations have a plan for how to respond to an identified mental health emergency (e.g., risk or suicide or harm to self/others).

A mental health emergency (sometimes called a mental health crisis) refers to any immediate event or situation in which an individual's behaviour/s puts them at risk of hurting themselves or others. It can include panic attacks, unsafe or unusual behaviours under the influence of drugs and/or alcohol, acute psychological distress, psychosis and/or psychotic-episodes, and aggressive and/or anti-social behaviours. Often, an individual is unable to resolve this situation with the skills and resources they have and may require assistance. Some of the most common mental health emergencies, and those that are also likely to be experienced in a sport context, are suicide (both attempts and death by suicide), and harm to oneself or others. Having a diagnosed mental health disorder, or symptoms of one is not considered a mental health crisis.

In 2021 alone, over 3,000 Australians died by suicide, with two-thirds of these deaths in boys and men [14]. Therefore, it is important that community sport organisations know what to do in the event of a mental health emergency occurring to ensure the safety of those at risk.

WHO FOR

At the very least, club committee members (including the club Mental Health Champion) and coaches/managers should know how to respond to a mental health emergency in their club or association. This plan will be important in for providing clear assistance and direction for responding to mental health emergencies that may pertain to all members at different levels of the club or only to specific members.

If a club wishes to do more, they can actively communicate and share this plan with all members of the club (e.g., players, parents, volunteers), such as via email.

4.6 Responding to a Mental Health Emergency

Responding to a Mental Health Emergency: Community sport organisations have a plan for how to respond to an identified mental health emergency (e.g., risk or suicide or harm to self/others).

WHO IS RESPONSIBLE

It is the responsibility of each club to have their own plan for how they will both identify and respond to a mental health emergency that may be affecting a member of their sport organisation. The club committee members, with assistance from the Mental Health Champion (see 4.8) should work together on developing a broad plan for their club. It is the responsibility of the Mental Health Champion to ensure the plan, just like the larger Mental Health Action Plan, is updated biennially or more frequently, if necessary.

The direct role that the club members and committee play in responding to a mental health emergency will differ depending on the emergency, the club and the individual experiencing the crisis. The 'first responder' for each club may also differ based on several factors. For example, if there is a trained mental health professional who is also a committee member, they may be in the best place to respond first. However, a particular coach may have a better relationship with the individual in crisis, making them the best person to respond first.

It is the responsibility of the district and regional associations to ensure an emergency plan is developed and readily accessible if needed. State and national sport organisations also have the responsibility to promote the importance of having such a plan in case of a mental health emergency.

HOW

Each club should have a documented plan for how they will identify and respond to a mental health emergency at their club. It needs to be readily accessible, and applicable to all club members, rather than having a single plan for one person. This will form a section of the Mental Health Action Plan (see 4.9).

4.6 Responding to a Mental Health Emergency

Responding to a Mental Health Emergency: Community sport organisations have a plan for how to respond to an identified mental health emergency (e.g., risk or suicide or harm to self/others).

Some key areas that should form part of the mental health emergency plan, and are included in the template, include:

- Potentially stressful situations or events that may trigger a mental health emergency
- Warning signs/symptoms of a mental health emergency
- What to do in a mental health emergency
- How to follow-up after a mental health emergency

It is important that club members are not only aware but also knowledgeable about how to enact this plan if an emergency were to occur. To assist in creating a mental health emergency plan, we have created a template for you that you can quickly and easily fill out. Some areas have already been filled out for you. This specific plan should be placed in a visible location so it can be referred to in the event of an emergency.

WHEN

In line with the Mental Health Action Plan (see 4.9), it will need to be updated biennially to ensure the contact information is up to date. If a mental health emergency is to occur, we recommend a review process of the plan is undertaken. All current committee members and coaches/managers need to be aware of the plan and how to enact it. Any incoming committee members or coaches must also ensure they are aware and up-to-date with the plan in the event of an emergency.

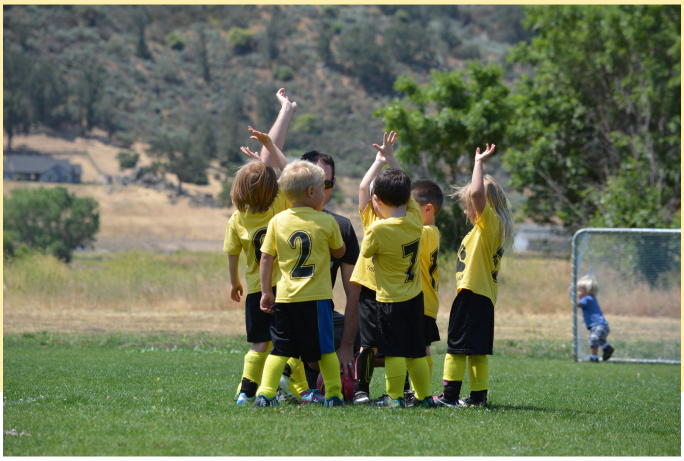

4.7 Responding to Major Events That May Impact Mental Health

Responding to Major Events that May Impact Mental Health: Community sport organisations have a plan for how to respond to major events or critical incidents that may impact the psychological wellbeing of their members.

When an incident occurs that involves a club member (current or previous), or a relative of a club member, it is important that the sport organisation has appropriate mechanisms in place to support those involved, or affected, to ensure no additional psychological harm is caused. A major event can be any incident that occurs inside or outside of the sport organisation, that directly influences one or more members.

Some examples of major events/critical incidents include:

- Serious physical injury on or off the field
- The death of a current/former club member or a relative/friend of a club member
- Members that are the victim of crime or have been implicated in one
- Natural disasters that affect the members and/or club
- Violent incidents affecting members

It is important that sport organisations have a pre-emptive plan and the appropriate mechanisms in place to ensure that if a major event does occur, the psychological wellbeing of those involved, directly and indirectly, is looked after both within the organisation and external to the organisation. There may be different supports available for different types of incidents.

WHO FOR

At the very least, the club committee members and the club Mental Health Champion should be aware and knowledgeable about this plan. If a major event were to take place, these individuals should know what to do to provide support or, where to access the appropriate support.

WHO IS RESPONSIBLE

All clubs need to ensure they have their own plan for if and when a major event may occur. It is the responsibility of the nominated club Mental Health Champion (see 4.8) and the club committee to prepare this plan.

4.7 Responding to Major Events That May Impact Mental Health

Responding to Major Events that May Impact Mental Health: Community sport organisations have a plan for how to respond to major events or critical incidents that may impact the psychological wellbeing of their members.

HOW

The Major Event Response Plan forms part of the larger Mental Health Action Plan (see 4.9). The plan will focus on what the club can do to provide support to members, from the short term into the long term, who may have witnessed or been affected by an incident. As part of this larger plan, you will be provided a guide and template that will assist in creating a response plan. It is important to remember that this plan is not a response to the event, but rather a response to the potential psychological distress as a result of the event.

Some sections of the plan provide additional information on major events/critical incidents and where you can seek specific support for a specific type of incident, whereas other sections provide a more general overview and then have space for clubs to enter the details of their own plan. We do not recommend engaging in processes such as Critical Incident Stress Management or Critical Incident Stress De-Briefing, as there is not enough evidence to show it does not cause more psychological harm [15].

WHEN

The Major Event Response Plan should be completed as soon as feasible. They will need to be updated biennially (every two years) to ensure the contact and resource information is up to date. This update can coincide with the update of other plans created as part of these guidelines. All current committee members need to be aware of the plan and how to enact it. Any incoming committee members must also ensure they are up-to-date with the plan.

If a major event/critical incident were to occur, the Major Event Response Plan would need to be reviewed and potentially updated to reflect any change in processes.

4.8 Implementing a Mental Health Champion

Implementing a Mental Health Champion: Sport clubs and organisations need to have a mental health officer who actively oversees and contributes to the implementation of the mental health guidelines.

According to Mental Health First Aid, a mental health officer is a "*designated person in an organisation who offers initial mental health support to others in the organisation who may be experiencing a mental health problem*". Similarly, a mental health champion is a nominated individual within an organisation that is the expert in everything related to mental health and wellbeing. In this instance, having a dedicated mental health champion in a sport club will allow for the current mental health guidelines to be actively advocated and monitored. By having an individual (or perhaps a small sub-group) hold this position within the sport organisation committee, it will ensure that mental health and wellbeing within the club is treated as an area of interest. Whilst everyone in a sports organisation has a role to play to ensure a psychologically safe environment, the mental health champion is the one who will oversee the guidelines.

By having a dedicated person within the committee who is responsible for many of these guidelines, it will ensure a streamlined process for updating plans, monitoring outcomes and ensuring all those involved are aware and knowledgeable.

WHO FOR

It is recommended that if a sport organisation already has a health and safety officer, or a member protection officer, on their committee, they combine this role with that of the mental health champion. However, an additional role on the committee can be created, if preferred.

Similarly, depending on the individual sports organisation, having a sub-committee share the role rather than one person, may be more suitable. However, it is important to keep in mind that having a small team can result in 'social loafing', where some team members put in less effort when they are in a team, compared to when working alone. A club may wish to appoint a 'leader' of the sub-committee to reduce this possibility.

4.8 Implementing a Mental Health Champion

Implementing a Mental Health Champion: Sport clubs and organisations need to have a mental health officer who actively oversees and contributes to the implementation of the mental health guidelines.

It is not recommended that the President, Vice-President or any coaches/managers take on this role due to potential conflicts of interest (e.g., a player may wish to provide confidential feedback about their coach to the mental health champion but cannot do so if their coach is also in this role).

WHO IS RESPONSIBLE

It is the responsibility of each sports club to ensure they have a mental health champion. The committee, led by the President, will be responsible for selecting an appropriate individual for this position, whether they are currently a member of the committee or recruited specifically for the role. It is also the responsibility of the committee, in particular the President, to ensure the appointed mental health champion receives (or is already certified) in Mental Health First Aid Training (see 4.4 for a link to their website).

At higher levels of sport, regional, state and national associations need to ensure they set the correct precedence and have a nominated mental health champion (or something similar). Higher level sport organisations are responsible for promoting the importance of a mental health officer/champion in sport.

HOW

Each sport club committee must appoint someone (or a sub-committee) who should be the mental health champion/s for the club. The role of the mental health champion/s is to:

- Oversee the implementation of the mental health guidelines.
- Actively promote mental health and wellbeing within the club, such as through club social events.
- Ensure the Mental Health Action Plan (see 4.9) is developed and available for all members of the club.
- Be knowledgeable in and action the club Mental Health Action Plan.

4.8 Implementing a Mental Health Champion

Implementing a Mental Health Champion: Sport clubs and organisations need to have a mental health officer who actively oversees and contributes to the implementation of the mental health guidelines.

- Be responsible for the updating of any mental health plans within the club.
- Actively organise and monitor the mental health literacy training, coach development training, and other mental health-related education within the club.
- Be the champion of mental health initiatives within the club.
- Be a role model for continuing and updating their mental health and wellbeing training
- Be the highest escalation point for any mental health or wellbeing concerns within the club.
  - The mental health champion does not have to be the first responder, or the one that other club members report to first. However, they should still be trained to appropriately be the first respond, if necessary.

A good mental health champion should be someone who:

- Has received Mental Health First Aid Training and is accredited (or will receive it early on in the role)
- Has received (or currently completing) a degree or certificate in mental health and wellbeing from a tertiary institution, such as TAFE or University (e.g., psychology, social work, counselling, occupational therapy)
- Is an advocate for mental health and wellbeing in the community
- Is willing to engage in additional mental health training (see Guideline 1) with other members of the committee
- Is willing to be the champion of mental health initiatives

For more information on what the role of a mental health officer or champion is in an organisation, please visit the Mental Health First Aid website.

4.8 Implementing a Mental Health Champion

Implementing a Mental Health Champion: Sport clubs and organisations need to have a mental health officer who actively oversees and contributes to the implementation of the mental health guidelines.

If you are unable to fill the role of mental health champion with someone with recommended attributes, the club should select the most appropriate person based on who is interested and willing. At the every minimum, the mental health champion must be an advocate for mental health and wellbeing, and already be accredited (or receive training as part of the role) in Mental Health First Aid.

WHEN

If a club does not already have a mental health champion, one should be appointed as soon as feasible. If a club does have one, the above roles and responsibilities should be shared with them as soon as is feasible. If a mental health champion were to resign from their role, another individual needs to be appointed to the role.

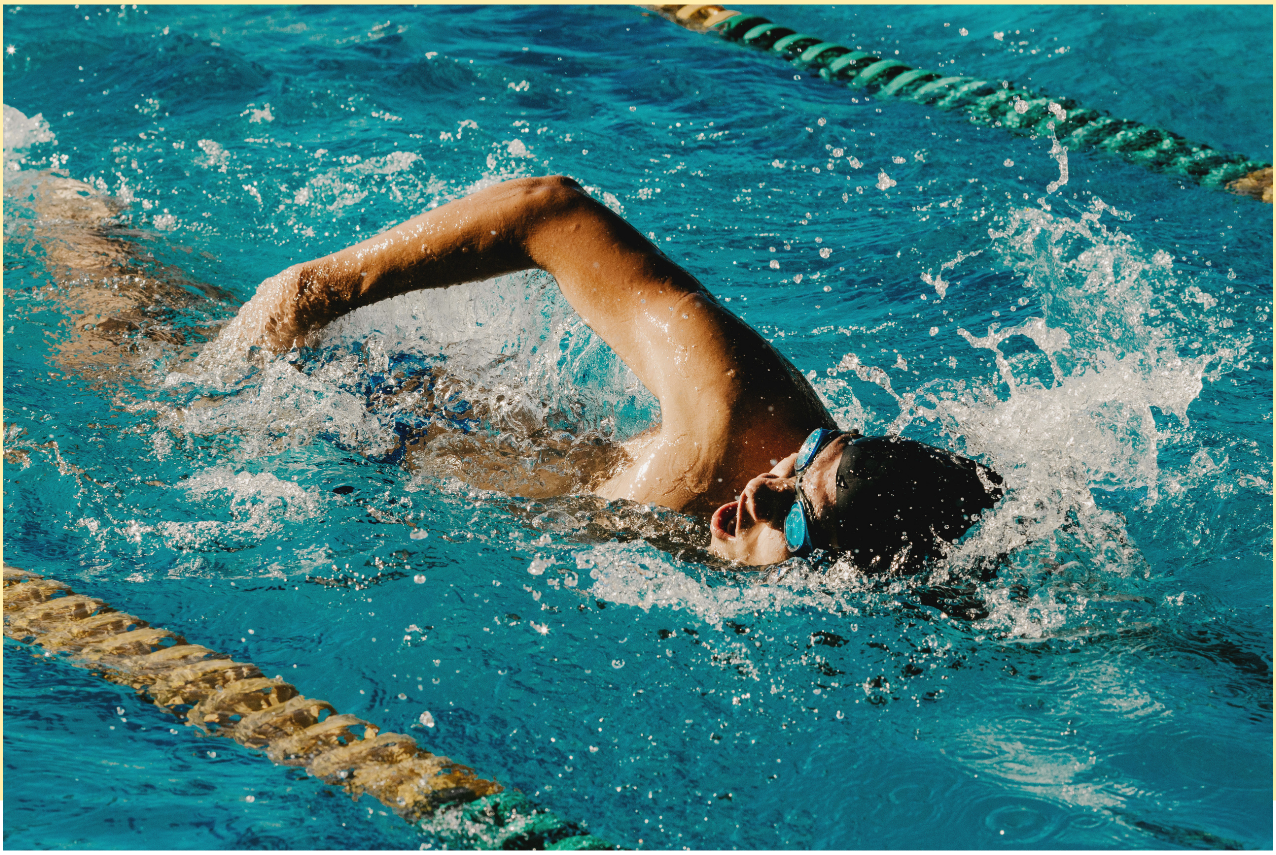

4.9 Developing a Mental Health Action Plan

Developing a Mental Health Action Plan: Community sport organisations have a completed up-to-date mental health action plan outlining how they will prevent, promote and respond to mental health and wellbeing in sport.

WHY

A Mental Health Action Plan provides an accessible overarching document to club committees and members on how the club/sport is implementing the current mental health guidelines. This plan also allows clubs to tailor the guidelines to their own resources and indicate the roles that everyone has to play in ensuring a mentally healthy environment. An Action Plan will also allow for committees and associations to track exactly what clubs are doing and how they are making healthy change.

WHO FOR

The Mental Health Action Plan is for all members and affiliates of a sport club. It should be actively shared with all current members at the start of each season, such as via email. The plan should also be accessible to all club members, either through being in an easily accessible location at the club or training ground, or on the website. In line with other recommendations within the guidelines, this plan should aim to prevent a mentally unhealthy sport environment, as well as promote and provide a positive mental health and wellbeing for all members (e.g., committee, coaches, players, officials, volunteers, and parents).

WHO IS RESPONSIBLE

The Mental Health Champion (see 4.8) at the club is responsible for ensuring the Mental Health Action Plan is developed, shared, accessible and regularly updated. It is also the responsibility of the Mental Health Champion to implement and monitor the implementation of the plan to ensure all aspects of the plan are being addressed. If a club has not yet appointed a Mental Health Champion, the members of the committee should then work together to create this plan. Whilst doing so, appointing a Mental Health Champion should also be a priority.

State and national sport organisations also have the responsibility to promote the importance of having such a Mental Health Action Plan, as mental health is just as important as physical health.

4.9 Developing a Mental Health Action Plan

Developing a Mental Health Action Plan: Community sport organisations have a completed up-to-date mental health action plan outlining how they will prevent, promote and respond to mental health and wellbeing in sport.

HOW

The Mental Health Action Plan should specify how the club plans to address each of the guidelines laid out in this document. To assist in this process, we have provided a Mental Health Action Plan template that we recommend using. It is quick and easy to use and can be filled out specifically with reference to the individual club, committee, and their available resources. This template also allows the club to update the plan with their own logos and branding. The completed plan should be actively shared with members and be readily available on the website for all members to access at any time.

WHEN

The Mental Health Action Plan should be reviewed and updated biennially. If necessary, the template and plan can be adapted and amended more regularly. It may be important that in the event of mental health emergency or major event/critical incident, that the plan is reviewed and updated. The club may consider consulting its members to gain feedback on the implementation of the plan.

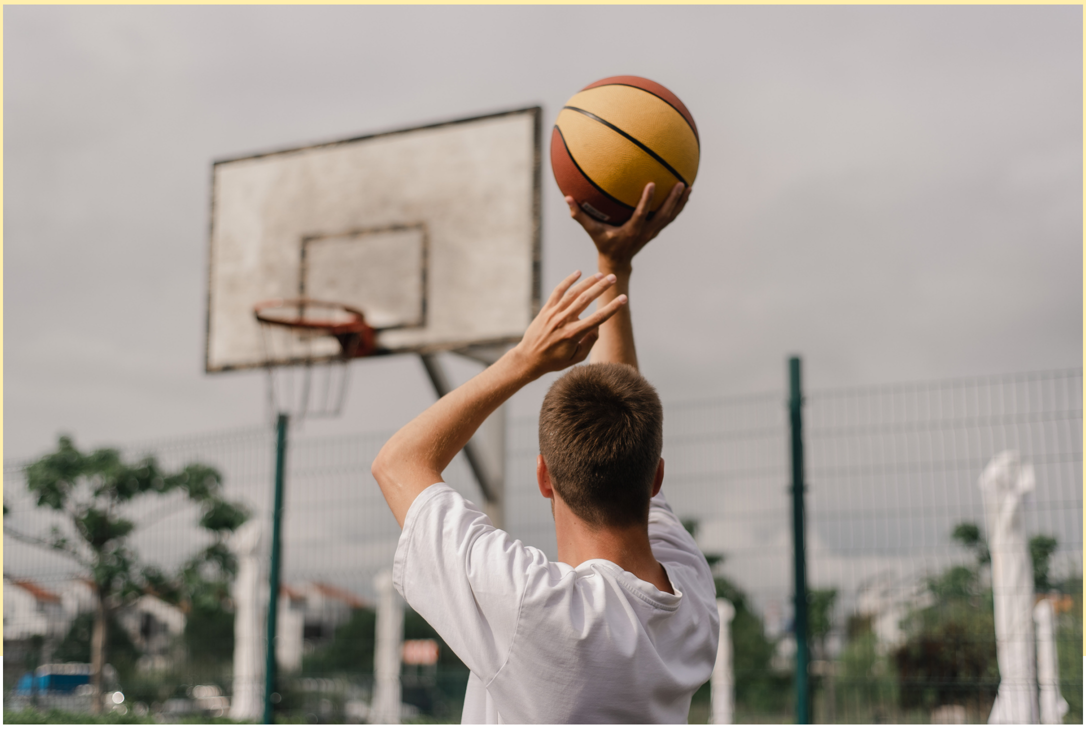

5. REFERENCES

1. Jorm AF, Korten AE, Jacomb PA, Christensen H, Rodgers B, Pollitt P. "Mental health literacy": a survey of the public's ability to recognise mental disorders and their beliefs about the effectiveness of treatment. Medical Journal of Australia. 1997;166:182–6.

2. Bapat S, Jorm A, Lawrence K. Evaluation of a Mental Health Literacy Training Program for Junior Sporting Clubs. Australasian Psychiatry. 2009;17:475–9.

3. Hurley D, Allen MS, Swann C, Vella SA. A Matched Control Trial of a Mental Health Literacy Intervention for Parents in Community Sports Clubs. Child Psychiatry Hum Dev [Internet]. 2021;52:141–53. Available from: https://doi.org/10.1007/s10578-020-00998-3

4. Vella SA, Swann C, Batterham M, Boydell KM, Eckermann S, Ferguson H, et al. An Intervention for Mental Health Literacy and Resilience in Organized Sports. Med Sci Sports Exerc. 2021;53:139–49.

5. Smith RA, Applegate A. Mental health stigma and communication and their intersections with education. Commun Educ. 2018;67:382–93.

6. Rüsch N, Angermeyer MC, Corrigan PW. Mental illness stigma: Concepts, consequences, and initiatives to reduce stigma. European Psychiatry. 2005;20:529–39.

7. Bowers H, Manion I, Papadopoulos D, Gauvreau E. Stigma in school-based mental health: perceptions of young people and service providers. Child Adolesc Ment Health. 2013;18:165–70.

8. Wo DXH, Ambrose ML, Schminke M. What Drives Trickle-Down Effects? A Test of Multiple Mediation Processes. Academy of Management Journal. 2015;58:1848–68.

9. Vella SA, Mayland E, Schweickle MJ, Sutcliffe JT, McEwan D, Swann C. Psychological safety in sport: a systematic review and concept analysis. Int Rev Sport Exerc Psychol [Internet]. 2022;1–24. Available from: https://doi.org/10.1080/1750984X.2022.2028306

10. Côté J, Gilbert W. An Integrative Definition of Coaching Effectiveness and Expertise. Int J Sports Sci Coach. 2009;4:307–23.

5. REFERENCES

11. Vargas-Tonsing TM. Coaches' Preferences for Continuing Coaching Education. Int J Sports Sci Coach. 2007;2:25–35.

12. Walton CC, Carberry S, Wilson M, Purcell R, Olive L, Vella S, et al. Supporting Mental Health in Youth Sport: Introducing a Toolkit for Coaches, Clubs, and Organisations. Int Sport Coach J. 2022;9:263–70.

13. Vella SA, Perlman DJ. Mastery, Autonomy and Transformational Approaches to Coaching: Common Features and Applications. Int Sport Coach J. 2014;1:173–9.

14. Australian Bureau of Statistics. Causes of Death, Australia, 2021. Canberra;

15. Kagee A. Concerns about the effectiveness of critical incident stress debriefing in ameliorating stress reactions. Crit Care. 2002;6:88.

16. Keyes CLM. The Mental Health Continuum: From Languishing to Flourishing in Life. J Health Soc Behav. 2002;43:207.

17. Pluhar E, McCracken C, Griffith KL, Christino MA, Sugimoto D, Meehan WP. Team Sport Athletes May Be Less Likely To Suffer Anxiety or Depression than Individual Sport Athletes. J Sports Sci Med. 2019;18:490–6.

18. Geidne S, Quennerstedt M, Eriksson C. The youth sports club as a health-promoting setting: An integrative review of research. Scand J Public Health. 2013;41:269–83.

19. Eime R, Young J, Harvey J, Charity M, Payne W. A systematic review of the psychological and social benefits of participation in sport for children and adolescents: informing development of a conceptual model of health through sport. International Journal of Behavioural Nutrition and Physical Activity. 2013;10.

6. RESOURCES AND TEMPLATES

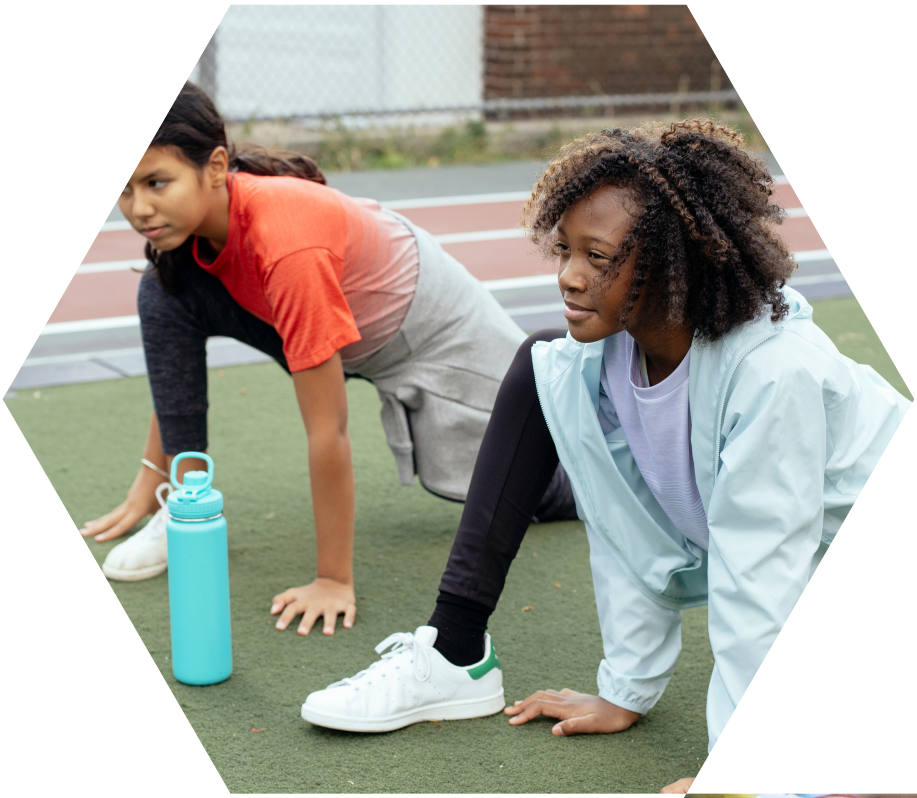

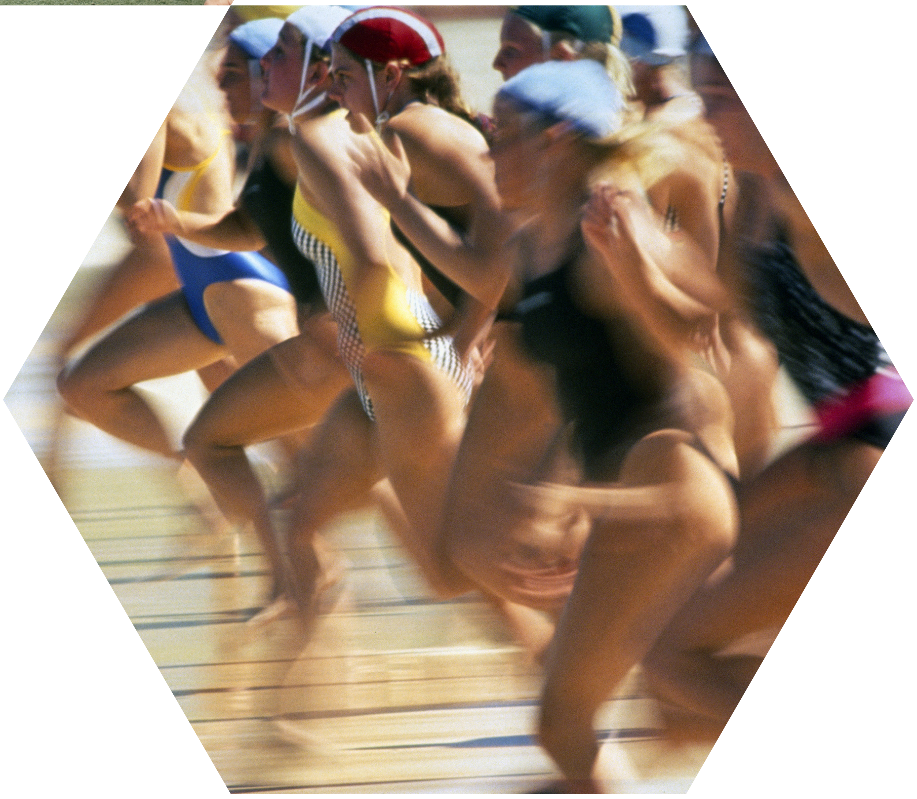

MENTAL HEALTH GUIDELINES FOR COMMUNITY SPORT

MENTAL HEALTH ACTION PLAN

SPORT CLUB NAME: ____

DATE OF COMPLETION: ______________________

PROMOTING WELLBEING

ACTIVITY	OBJECTIVES	TIMEFRAME	RESOURCES	WHO INVOLVED

MENTAL HEALTH ACTION PLAN

REDUCING MENTAL HEALTH STIGMA

ACTIVITY	OBJECTIVES	TIMEFRAME	RESOURCES	WHO INVOLVED

MENTAL HEALTH ACTION PLAN

PROMOTING COACH DEVELOPMENT

ACTIVITY	OBJECTIVES	TIMEFRAME	RESOURCES	WHO INVOLVED

HOW WILL YOU MONITOR THE PROGRESS AND/OR COMPLETION OF TRAINING?

MENTAL HEALTH ACTION PLAN

INCREASING MENTAL HEALTH LITERACY

ACTIVITY	OBJECTIVES	TIMEFRAME	RESOURCES	WHO INVOLVED

HOW WILL YOU MONITOR THE PROGRESS AND/OR COMPLETION OF TRAINING?

MENTAL HEALTH ACTION PLAN

LOCATING APPROPRIATE SUPPORT

ACTIVITY	OBJECTIVES	TIMEFRAME	RESOURCES	WHO INVOLVED
Complete 'mental health support options' template	To ensure we are prepared if/when a member of the club needs assistance	Complete by mid-season. Updated every 2 years (or after emergency/major event)	Use associated template	Mental Health Champion and/or committee to complete

MENTAL HEALTH ACTION PLAN

RESPONDING TO A MENTAL HEALTH EMERGENCY

ACTIVITY	OBJECTIVES	TIMEFRAME	RESOURCES	WHO INVOLVED
Complete the 'mental health emergency plan'	To ensure we are prepared if/when a mental health emergency arises	Complete by mid-season. Updated every 2 years (or after emergency/event)	Use associated template	Mental Health Champion and/or committee to complete

MENTAL HEALTH ACTION PLAN

RESPONDING TO MAJOR EVENTS THAT MAY IMPACT MENTAL HEALTH

ACTIVITY	OBJECTIVES	TIMEFRAME	RESOURCES	WHO INVOLVED
Complete the 'major events response plan'	To ensure we are prepared if/when a major event occurs	Complete by mid-season. Updated every 2 years (or after major event)	Use associated template	Mental Health Champion and/or committee to complete

MENTAL HEALTH ACTION PLAN

IMPLEMENTING A MENTAL HEALTH CHAMPION

ACTIVITY	OBJECTIVES	TIMEFRAME	RESOURCES	WHO INVOLVED
Identify an appropriate person for the role	To ensure the individual is the best person for the role	As soon as possible		Committee
Ensure individual is appropriately trained	To ensure they are readily prepared for the role	As soon as possible		Committee and nominated individual

MENTAL HEALTH SUPPORT OPTIONS

MENTAL HEALTH EMERGENCY PLAN

MAJOR EVENTS RESPONSE PLAN

MENTAL HEALTH SUPPORT OPTIONS

ONLINE & PHONE MENTAL HEALTH SUPPORT

- **BeyondBlue** | https://www.beyondblue.org.au/ | 1300 22 4636
- **Headspace** | https://headspace.org.au/
- **Lifeline Australia** | https://www.lifeline.org.au/ | 13 11 14 !
- **Suicide Call Back Service** | https://www.suicidecallbackservice.org.au/ | 1300 659 467 !
- **Head to Health** | https://www.headtohealth.gov.au/ | 1800 595 212

LOCAL COMMUNITY MENTAL HEALTH SUPPORT

Completed by:_____________________________

Signature: ________________________________
Position:_______________________
Date:_________________

Next review date:_________________

MENTAL HEALTH EMERGENCY PLAN

If someone has attempted, or is at immediate risk of attempting to harm themselves or someone else, call Triple Zero (000) immediately.

The goal of responding to a mental health emergency is de-escalation.

SITUATIONS/EVENTS THAT MAY TRIGGER A MENTAL HEALTH EMERGENCY

- Death of a loved one
- Losing employment or failing a class/course
- Changes in relationships (e.g., a divorce or breakup)
- Switching or stopping mental health treatments
- Discrimination
- Natural disaster, violence, or terrorism
- Large economic losses
- Medical diagnosis

WARNING SIGNS/SYMPTOMS OF A MENTAL HEALTH EMERGENCY

- Hallucinations, such as hearing or seeing things that aren't there
- Bizarre or unusual thinking
- Paranoia
- Non-suicidal self-injury (deliberate self-harm)
- Threatening behaviour/s
- Destructive or high-risk behaviour/s
- Confusion and disorientation
- Expressing anger and/or rage
- Talking directly about death, suicide, not wanting to live anymore, making threats to end their life, or have made a plan to suicide

WHAT NOT TO DO DURING A MENTAL HEALTH EMERGENCY

- Blame them
- Tell them to 'snap out of it' or 'get over it'
- Be hostile or sarcastic
- Get over-involved or over-protective
- Trivialise their experience (e.g., don't tell them to smile or get their act together)
- Belittle or dismiss their feelings
- Be patronising
- If suicidal, do not leave them alone

MENTAL HEALTH EMERGENCY PLAN

WHAT TO DO DURING A MENTAL HEALTH EMERGENCY

1. **Stay calm**. Speaking calmly without raising your voice can help avoid causing further panic. You'll also want to avoid arguing with the person or overreacting.

2. **Listen.** Listening can help you figure out what they need and understand their current symptoms.

3. **Ask how you can help**. Expressing support and concern can help them feel like they're not alone, especially if you're not with them in person. See what you can do for them, but understand that they may not be in a state of mind to know what they need, so you may need to be specific. For example, you might ask, "*Do you need me to call someone for you?*" or "*Do you need a ride to the hospital?*"

4. **Offer them options.** Though you may feel as if you need to take control of the situation, someone might get overwhelmed by that. So instead, you'll want to offer them options and be patient.

5. **Give them space.** You don't want them to feel trapped or boxed in. You'll also want to avoid touching them until they give you permission. Do not be too 'up in their face'.

- If at any point you feel unsafe, remove yourself from the situation immediately.
- If there is a physical injury, call 000 if it is life-threatening, and/or use First Aid Skills to attend to it there and then.

HOW TO FOLLOW-UP AFTER A MENTAL HEALTH EMERGENCY

- Look after yourself first. Take some time for self-care and reflection, and be sure to reach out for support if you need it.
- Report the event to any other bodies and/or parties, if required. Please follow your regional guidelines for events such as this.
- After a few days, check in with the person either in person or via a social media platform. This will show them you are there to support them and listen if they need.
- Encourage regular attendance at mental health support and treatment sessions. If there are limited options in your area, offer some accessible resources they can use. But do not force.
- Honour their experience; do not assume how they are thinking or feeling, ask them openly.

Repurposed from Mental Health First Aid (https://www.betterhealth.vic.gov.au/health/healthyliving/Mental-health-first-aid)

MENTAL HEALTH EMERGENCY PLAN

OTHER CONSIDERATIONS/NOTES/KEY STEPS FOR YOUR SPORTS ORGANISATION

FURTHER GUIDELINES TO ASSIST IN EMERGENCIES

Mental Health First Aid - Depression Guide
Mental Health First Aid - Panic Attacks Guide
Mental Health First Aid - Psychosis Guide
Mental Health First Aid - Non-Suicidal Self-Injury Guide

Completed by:_____________________________

Signature: _________________________________

Position:______________________

Date:_________________

Next review date:_________________

MAJOR EVENTS RESPONSE PLAN

CHAIN OF COMMAND & KEY DETAILS

Who will be responsible for management and coordination?

Name: ____

Contact number: ________________________________

Who will be responsible for all communication?

Name: ____

Contact number: __________________________________

Who will be responsible for media communication?

Name: ____

Contact number: ___________________________________

What will be the mutual location for people to gather?

____

Who are some senior club members who can provide support?

Name: ____

Contact number: ___________________________________

Name: ____

Contact number: ___________________________________

What resources/support will you provide to members?

____

____

MAJOR EVENTS RESPONSE PLAN

CHECKLIST TO HELP YOU MANAGE YOUR REPSONSE

IMMEDIATELY AFTER

- [ ] If the incident has happened at the sports organisation, ensure the immediate safety of club members and visitors
- [ ] Identify an individual responsible for the management and coordination of the event
- [ ] Identify a central point of communication (who, how)
- [ ] If the incident has happened away from the sports organisation: Find out as many of the facts and circumstances as possible. Do not ignore rumours – investigate them immediately. Confirm facts with the family and/or police.
- [ ] Inform relevant individuals and bodies of the major event

THE FIRST 24 HOURS

- [ ] Draft a letter/email to communicate with members of the club via direct contact and media about the major event, and what the organisation is doing
- [ ] Reach out and liaise with the affected individuals and provide support
- [ ] Provide a place for club members to gather and talk, such as the clubhouse
- [ ] Direct members to access psychological support services (refer to Mental Health Support Options Template)

THE FIRST WEEK

- [ ] Facilitate a way for people and club members to send letters of support
- [ ] Create a website where people can make donations to the affected individuals
- [ ] Monitor the wellbeing of club members and link them with appropriate support services
- [ ] Hold an organisation or community-wide 'meeting' to discuss the major event and support that is available
- [ ] Consider the return to competition and what is important at this time

THE FIRST MONTH to SIX MONTHS

- [ ] Organise and ensure regular communication with club members about the event
- [ ] Continue to monitor the wellbeing of club members and link them with appropriate support services
- [ ] If a death has occurred, consider and plan for a memorial at the club, and future commemorations, birthdays, and anniversaries

MENTAL HEALTH GUIDELINES FOR COMMUNITY SPORT

MAJOR EVENTS RESPONSE PLAN

OTHER CONSIDERATIONS/NOTES/KEY STEPS FOR YOUR SPORTS ORGANISATION

Mental Health Support after a Natural Disaster - Phoenix Australia Guide
Mental Health Support After the Suicide of a Teammate - Guide

Completed by:______________________________

Signature: _________________________________

Position:_______________________

Date:_________________

Next review date:_________________

MAJOR EVENTS RESPONSE PLAN

EXAMPLE SCRIPT TO SEND TO CLUB MEMBERS

Support Service for __________________ Club members

We are all in shock from the untimely death of ____________________. To lose a loved one like, a dear friend and team mate, is one of the most difficult life experiences you will have to face. When the death is sudden and tragic, family and friends must cope with the sadness of their loss plus all their additional heightened feelings like confusion, questioning of self, anger and coming to terms with his death.

Should you wish to speak to someone in confidence about how you feel or if you need help or guidance to come to terms with death, please call: ___________________. If you or someone you know is in danger, distress or despair, call the emergency services on 000.

The above is a confidential service available to you and we encourage you to use it and call or message, if you need to talk to someone. Equally, should you know of any of your friends or colleagues, who are struggling to come to terms with __________________ death please encourage them to call also, or talk to a loved one about their feelings.

We also ask you to keep an eye out for each other, not to be shy or embarrassed about asking for help and to talk to and support each other during what is a very difficult time for us all. If there is anything we can do to help and support you please let us know. The clubhouse will be open every day 3pm-8pm if you wish to come down and chat with other members, and provide support to one another.

We will get through this tragic time together.

__________________________,
President,
on behalf of the __________________ Club Committee.

The above example script is just that, an example. Please edit the letter however you please, and however is most appropriate given the circumstances. You may also wish to include additional steps the club is taking to provide support.

Repurposed from Athletics Ireland (https://www.athleticsireland.ie/downloads/other/AAI_Critical_Incident_Response_Plan.pdf)

DISCLAIMER

The information in this guide is for general use only. While the research team has made every effort to ensure the information in this guide is accurate, the advice within it may not apply to all circumstances or all sport organisations. The research team is not responsible for the suitability of the information for your sport organisation's specific circumstances, or any actions taken as a result of the information included in this guide. You must make your own assessment of the information contained in this document and whether or not you choose to rely on it.

ACKNOWLEDGEMENTS

This resource was prepared by Dr Caitlin Liddelow from the Global Alliance for Mental Health for Sport at the University of Wollongong, in collaboration with Associate Professor Stewart Vella, Dr Matthew Schweickle and Dr Jordan Sutcliffe.

Many thanks to the Australian Research Council for funding this project, and the guideline development committee for their expertise and contributions.

ENQUIRIES

Please direct all enquiries regarding this document to Dr Caitlin Liddelow at caitlinl@uow.edu.au.

UNIVERSITY OF WOLLONGONG AUSTRALIA

Australian Government
Australian Research Council
